# cGAS-ISG15-RAGE axis reprogram necroptotic microenvironment and promote lymphatic metastasis in head and neck cancer

**DOI:** 10.1186/s40164-024-00531-5

**Published:** 2024-06-26

**Authors:** Jingyuan Li, Jun Tan, Tao Wang, Shan Yu, Guangliang Guo, Kan Li, Le Yang, Bin Zeng, Xueying Mei, Siyong Gao, Xiaomei Lao, Sien Zhang, Guiqing Liao, Yujie Liang

**Affiliations:** 1 Hospital of Stomatology, Guanghua School of Stomatology, Sun Yat-Sen University, Guangzhou, Guangdong People’s Republic of China; 2 Guangdong Provincial Key Laboratory of Stomatology, Sun Yat-Sen University, Guangzhou, Guangdong People’s Republic of China; 3 Department of Oral and Maxillofacial Surgery, Hospital of Stomatology, Sun Yat-Sen University, 56th Lingyuanxi Road, Guangzhou, 510055 Guangdong China

**Keywords:** Necroptosis, Head and neck squamous cell carcinoma (HNSCC), ISG15, Damage-associated molecular patterns (DAMPs), cGAS-STING

## Abstract

**Background:**

Cancer cells frequently evolve necroptotic resistance to overcome various survival stress during tumorigenesis. However, we have previously showed that necroptosis is widespread in head and neck squamous cell carcinoma (HNSCC) and contributes to tumor progression and poor survival via DAMPs-induced migration and invasiveness in peri-necroptotic tumor cells. This implicated an alternative strategy that cancers cope with necroptotic stress by reprogramming a pro-invasive necroptotic microenvironment (NME). Here, we aim to decipher how necroptotic cells shape the NME and affect HNSCC progression.

**Methods:**

Both our pre-established cellular necroptotic model and newly established Dox-induce intratumoral necroptosis model were used to investigate how necroptosis affect HNSCC progression. Transcriptomic alterations in peri-necroptotic tumor cells were analyzed by RNA-seq and validated in the NME in mice and patients’ samples. The differential DAMPs compositon among apopotosis. Necrosis, and necroptosis were analyzed by label-free proteomic technique, and the necroptosis-specific DAMPs were then identified and validated. The potential receptor for ISG15 were simulated using molecular docking and further validated by in vitro assays. Then the ISG15-RAGE axis was blocked by either knockdown of necroptotic-ISG15 release and RAGE inhibitor FPS-ZM1, and the impact on tumor progression were tested. Last, we further tested our findings in a HNSCC-patients cohort.

**Results:**

Necroptosis played a crucial role in driving tumor-cell invasiveness and lymphatic metastasis via tumor-type dependent DAMPs-releasing. Mechanistically, necroptotic DAMPs induced peri-necroptotic EMT via NF-κB and STAT3 signaling. Furthermore, intrinsic orchestration between necroptotic and cGAS-STING signaling resulted in producing a group of interferon stimulated genes (ISGs) as HNSCC-dependent necroptotic DAMPs. Among them, ISG15 played an essential role in reprogramming the NME. We then identified RAGE as a novel receptor for extracellular ISG15. Either blockage of ISG15 release or ISG15-RAGE interaction dramatically impeded necroptosis-driven EMT and lymphatic metastasis in HNSCC. Lastly, clinicopathological analysis showed high ISG15 expression in NME. Extensive necroptosis and high tumor-cell RAGE expression correlated with tumor progression and poor survival of HNSCC patients.

**Conclusions:**

Our data revealed a previously unknown cGAS-ISG15-RAGE dependent reprogramming of the necroptotic microenvironment which converts the necroptotic stress into invasive force to foster HNSCC-cell dissemination. By demonstrating the programmatic production of ISG15 via necroptosis-cGAS orchestration and its downstream signaling through RAGE, we shed light on the unique role of ISG15 in HNSCC progression. Targeting such machineries may hold therapeutic potential for restoring intratumoral survival stress and preventing lymphatic metastasis in HNSCC.

**Graphical Abstract:**

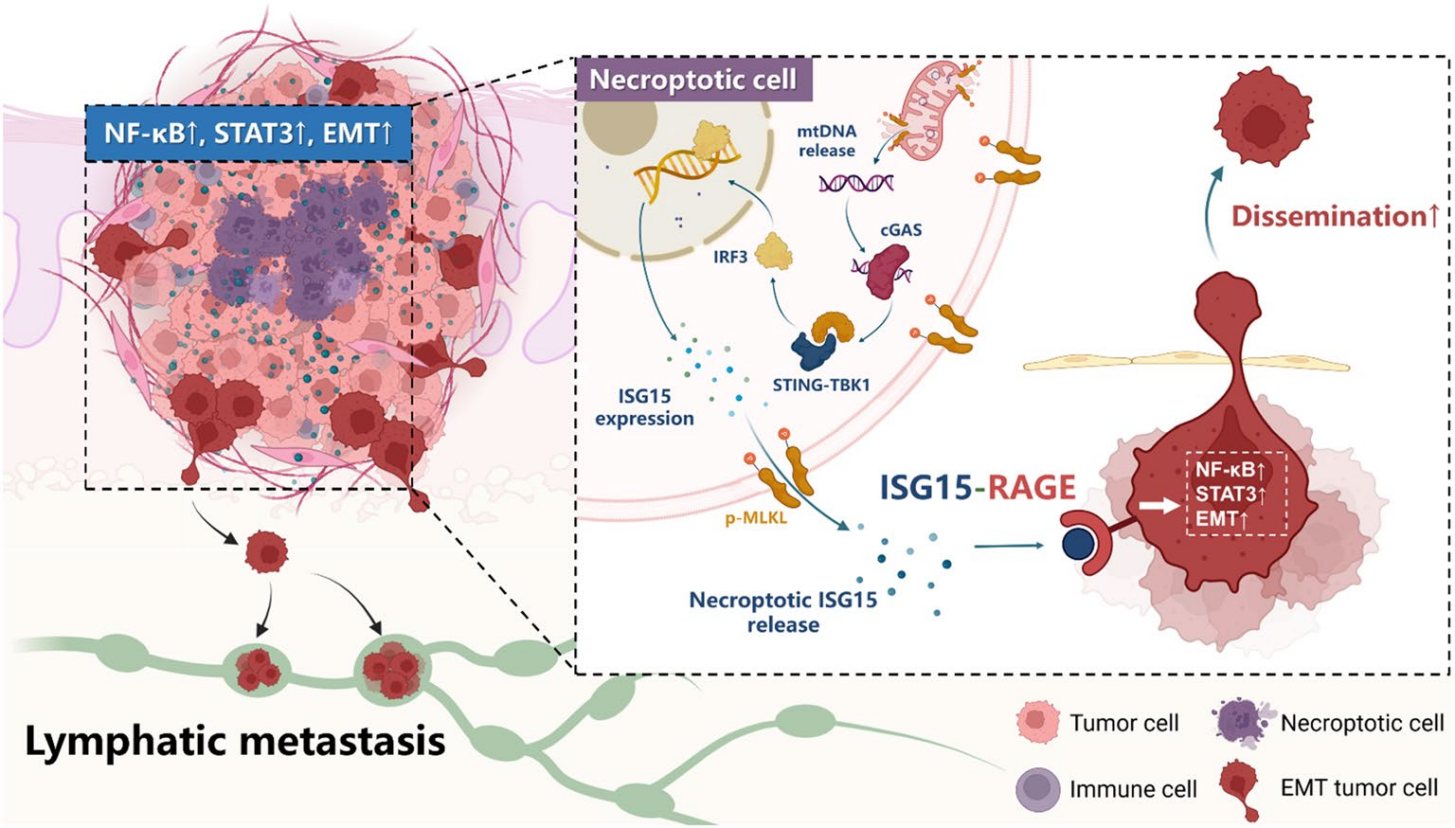

**Supplementary Information:**

The online version contains supplementary material available at 10.1186/s40164-024-00531-5.

## Background

Necroptosis is a regulated necrotic cell death which can be triggered in response to various cellular stress, damage, and infections and is considered crucial to restricting pathogenesis and maintenance of homeostasis [1–3]. Resisting cell death is a hallmark of cancer [4]. Tumor cells evolve many strategies to survive cell deaths induced by various physiological stress during tumorigenesis, including necroptosis [1, 4, 5]. Moreover, necroptosis itself poses survival stress to tumor cells as it has been reported to suppress tumorigenesis and induce antitumor immunity and immunosurveillance via necroinflammation [6–10]. Tumor cells developed various machineries including oncogene-driven, epigenic, transcriptional, and post-translational regulation of RIP3 and MLKL to form necroptotic resistance [11–16]. Consequently, loss/downregulation of RIP3 and MLKL expression are seen in a variety of cancer types and cancer cell lines which correlated with poor survival rate [1, 17–20]. Accordingly, inducing necroptosis is shown to overcome apoptotic resistance and enhance sensitivity to anticancer therapy [21–23]. Contradictorily, however, we previously reported that necroptosis is widespread and predominantly contributes to the tumor necrosis in head and neck squamous cell carcinoma (HNSCC), and extensive necroptosis correlates with tumor progression and poor prognosis [24]. Moreover, HNSCC-derived necroptotic DAMPs induced an invasive phenotype in peri-necroptotic HNSCC cells, while colorectal cancer-derived DAMPs failed to do so [24]. Our findings implicated an alternative strategy in coping with necroptotic stress: instead of evading necroptosis, cancers utilize certain machineries, which are seemingly tumor-type specific, to shape a pro-invasive necroptotic microenvironment. Yet such machineries remain largely unknown.

Head and neck squamous cell carcinoma (HNSCC) accounts for over 90% of head neck cancer, which is the seventh most prevalent cancer worldwide [25]. HNSCC exhibits a significant tendency for lymphatic metastasis, which is an essential indicator for advanced stage and poor survival [26, 27]. Deciphering the complex tumor biology in developing lymphatic metastasis holds therapeutic significance in HNSCC.

ISG15 is the first-described ubiquitin-like protein modifier which is induced by type I interferon signalling [28]. ISG15 canonically acts as a post-translational protein modifier through an enzymatic cascade-mediated conjugation process named ISGylation. Besides, ISG15 is also reported to be secreted and exert cytokine-like functions, yet the mechanisms regarding its release and functional routes remain elusive [28, 29]. Interestingly, ISG15 has been recognized by multiple previous studies as one of the highly upregulated genes and potential biomarkers in head and neck cancer [30–32], but how it is involved in HNSCC tumorigenesis is largely unclear. Herein, we showed that ISG15 was a crucial mediator for necroptosis-driven metastasis and was produced through an intrinsic orchestration of necroptotic and cGAS-STING signaling. Additionally, RAGE was identified as a novel receptor for extracellular ISG15 which mediated the ISG15-driven reprogramming of peri-necroptotic microenvironment and lymphatic metastasis. Our data represent a novel cGAS-ISG15-RAGE dependent machinery by which HNSCC cells convert the necroptotic stress into invasive force to facilitate their dissemination. Targeting such machineries may hold therapeutic potential for restoring intratumoral survival stress and preventing lymphatic metastasis in HNSCC.

## Results

### Intratumoral necroptosis facilitates lymphatic metastasis of HNSCC

**Fig. 1:**
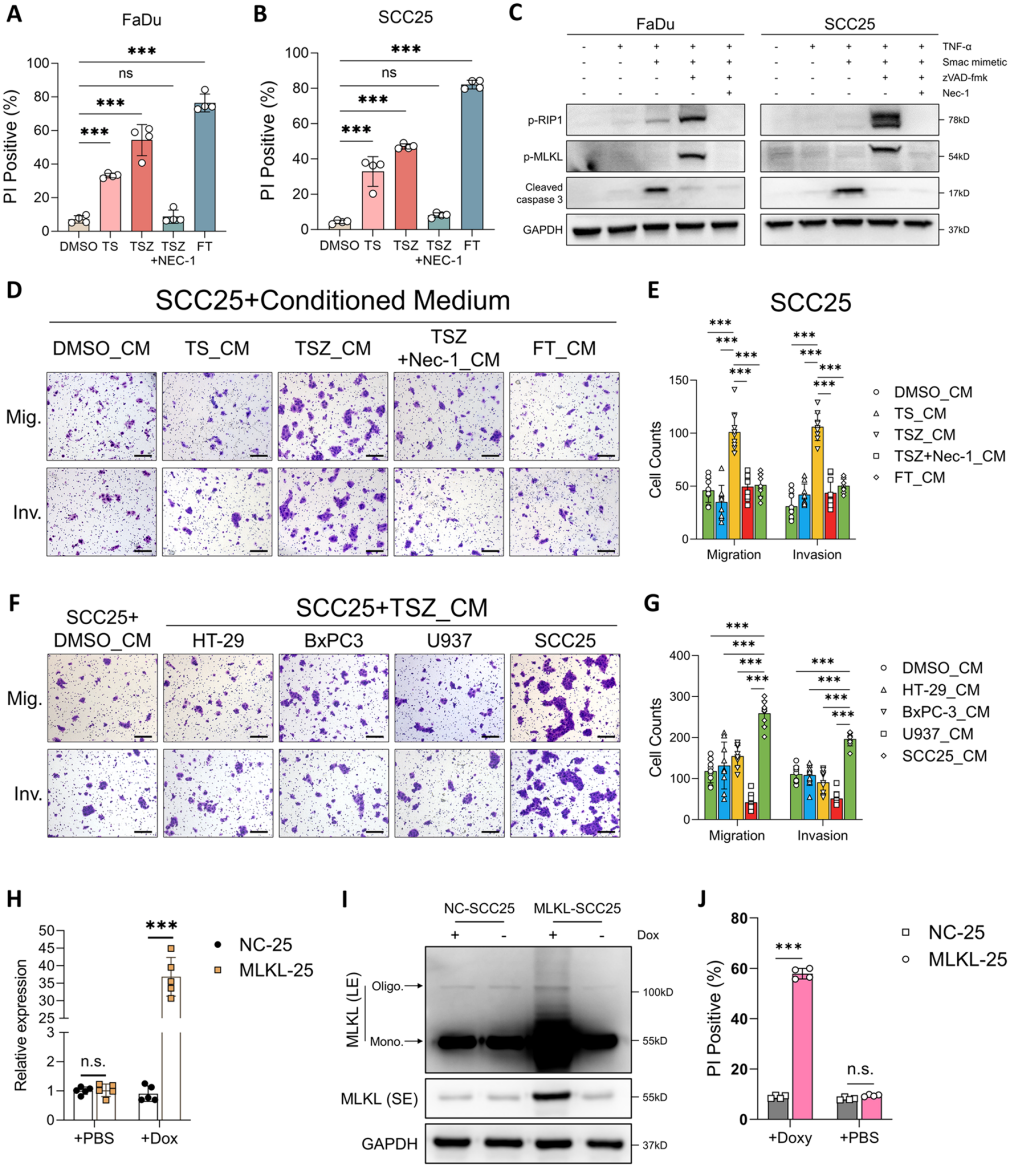
HNSCC-specific necroptotic DAMPs drives peri-necroptotic invasiveness. **A**–**C** SCC25 and FaDu cells were exposed to the indicated treatments for 24 and 48h, respectively. The cell death rates **A**, **B** were measured by PI staining, and the protein expression (**C**) were detected by western blotting. **D**, **E** Conditioned medium (CM) derived from TS (apoptosis), TSZ (necroptosis), FT (passive necrosis), TSZ + Nec-1 (inhibited necroptosis) and DMSO (vehicle control) treatments were collected and used to further treat SCC25 cells for 24 h. Tumor-cell migration and invasion were analyzed by Transwell assays (scale bar = 200μm, n = 9). **F**, **G** SCC25 cells were treated by necroptotic DAMPs derived from HT-29, BxPC3, U937 and SCC25 cells, or CM derived from DMSO treated cells. Tumor-cell migration and invasion were analyzed by Transwell assays (scale bar = 200μm, n = 9). MLKL-25 cells were induced by 1ug/ml Doxycycline for 8 h, the expression of MLKL were detected by qRT-PCR (**H**) and Western blotting (**I**). NC-25 and MLKL-25 were induced by Dox for 12 h, PI staining was used to measure cell death rate (**J**). *p < 0.05, **p < 0.01, ***p < 0.001. *LE* long exposure, *SE* short exposure

**Fig. 2:**
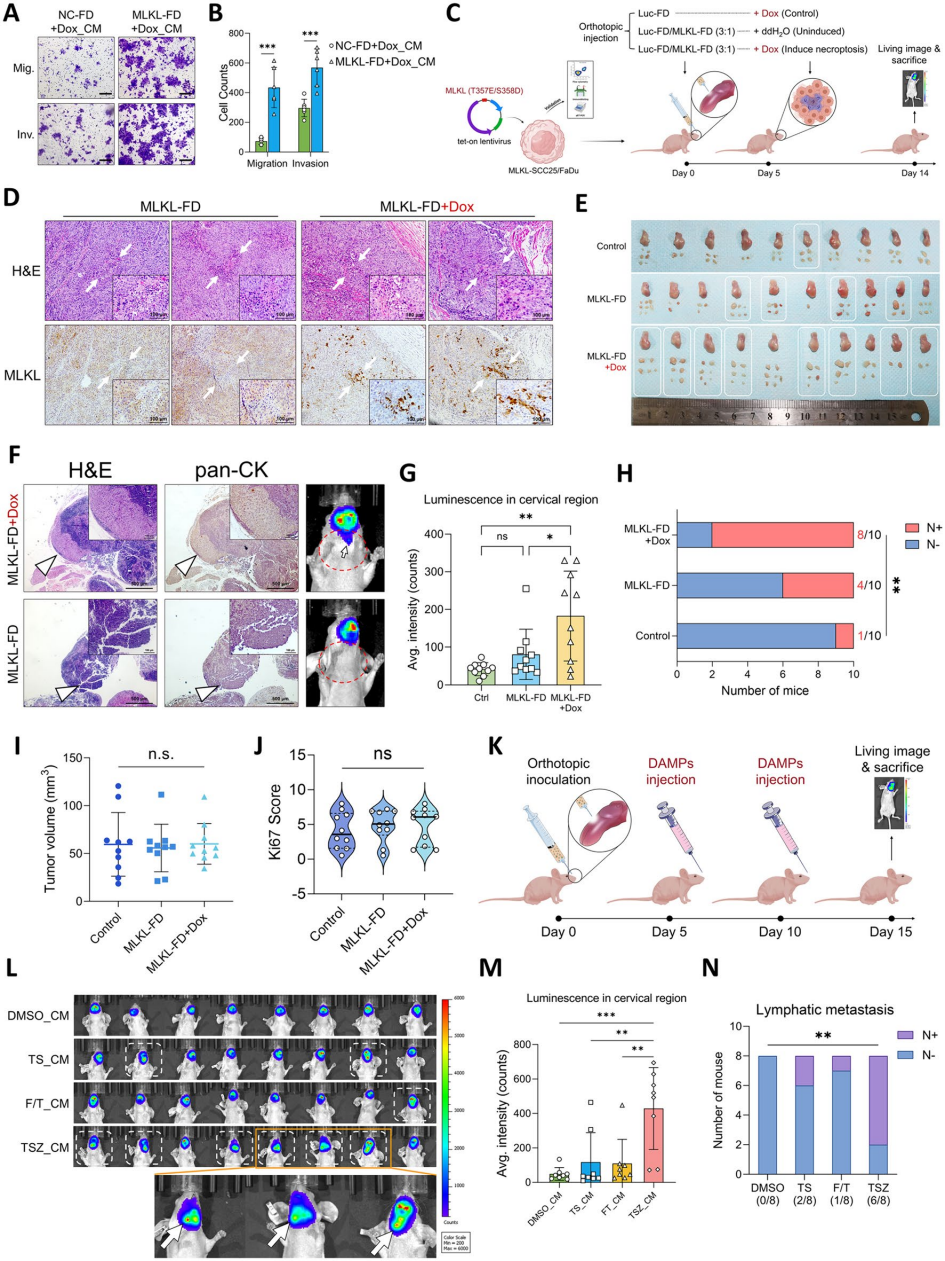
Necroptosis facilitates lymphatic metastasis of HNSCC via releasing DAMPs. **A**, **B** Transwell migration and invasion assays of FaDu cells treated by Dox-induced necroptotic DAMPs (scale bar = 200 μm, n = 6). **C** Schematic of the Dox-induced intratumoral necroptosis model. **D** H&E and MLKL staining of serial-sectioned primary tumors from Dox-induced and uninduced group. **E** Tongue and cervical lymph nodes collected at the endpoints. White frame indicated the mice with lymphatic metastasis which were confirmed by live imaging and pathological staining. **F** Representative image of HE and pan-CK stained metastatic and non-metastatic lymph node (left panel) and corresponding in vivo image (right panel, IVIS system, PerkinElmer, USA). **G** Statistical comparison of the Bio-luminescent signal in the cervical region among three groups. **H** Comparison of the lymphatic metastatic rate among three groups (Fisher’s exact test). **I**, **J** Statistical comparison of the volume and the Ki67 staining level of the xenograft tumor among three groups. **K** Schematic of intratumoral DAMPs injection model. **L** In vivo imaging analysis of cervical metastasis. Typical metastatic images were enlarged in the bottom panel, white arrow indicated the metastatic node. (**M**) Statistical comparison of the Bio-luminescent signal in the cervical region among four groups. **N** Statistical analysis of cervical metastatic rate in DAMPs injection model (Fisher’s exact test). n.s., not significant, *p < 0.05, **p < 0.01, ***p < 0.001

In our previous study, we established a cellular model of necroptosis using TNF-α + Smac mimetic + zVAD-fmk (TSZ) methods in HNSCC cells and demonstrated that necroptotic DAMPs promotes non-necroptotic-cell migration and invasion in vitro [24]. Herein, we first validated these findings. Consistent with our previous report, both TS, TSZ treatment, and freeze–thaw cycles (FT), which is a classic way to induce passive necrosis, can all significantly induce HNSCC-cell death (Fig. 1A, B). TS treated cells showed upregulated cleaved caspase 3, suggesting an apoptotic cell death (Fig. 1C). In contrast, TSZ treatment induced significant phosphorylation of RIP1 and MLKL while in the absent of cleasved caspase 3 (Fig. 1C), which indicated the activation of necroptotic signaling. Moreover, the necroptotic inhibitor Nec-1 can dramatically abolish TSZ-induced necroptosis (Fig. 1A–C). Next, HNSCC cells were treated with DAMPs collected from apoptotic, necrotic and necroptotic cells, respectively. And consistent with our previous findings, cells treated with necroptotic DAMPs showed dramatically increased migratory and invasive capacity (Fig. 1D, E and Fig. S1A, B). Our previous data also indicated that this DAMPs-induced invasiveness may be tumor-type specific as necroptotic DAMPs derived from colorectal cancer cell line HT-29 failed to promote the migration and invasion of HNSCC cells [24]. We validated this hypothesis here by showing that necroptotic DAMPs derived from colorectal cancer, pancreatic cancer and histiocytic lymphoma were unable to promote HNSCC-cell migration and invasion (Fig. 1F, G and Fig. S1C, D). Next, to investigated how necroptosis affects HNSCC progression in vivo, we transfected SCC25 and FaDu cells with tet-on lentivirus (Fig. S1E) containing phospho-mimetic MLKL (T357E/S358D) [33] and generated two doxycycline-inducible necroptosis cell lines (MLKL-25, MLKL-FD). In vitro validation showed that Dox induced MLKL expression, oligomerization, and cell death in MLKL-25 and MLKL-FD cells (Fig. 1H–J and Fig. S1F-H). DAMPs derived from Dox-induced necroptosis can also promote tumor-cell migration and invasion. (Fig. 2A, B and Fig. S1I, J).

We then designed an in vivo assay to investigate how intratumoral necroptosis affects the cervical lymphatic metastasis of HNSCC (Fig. 2C). Immunohistochemical analysis showed prevalent MLKL-overexpressed (MLKL^high^) clusters in the Dox-treated group while they were absent in Dox (-) groups (Fig. 2D). These MLKL^high^ clusters colocalized with necrotic foci on HE sections (Fig. 2D), which validated that the intratumoral necroptosis were induced by Dox. Live imaging plus pathological analysis identified a significantly higher metastatic rate in Dox-treated group (Fig. 2E–H and Fig. S1L), suggesting that intratumoral necroptosis facilitates lymphatic metastasis in vivo. Notably, consistent with previous in vitro finding that necroptotic DAMPs did not affect tumor-cell proliferation, both the tumor volume and the Ki67 level did not show significant differences among all groups (Fig. 2I, J and Fig. S1K). Next, we used an intratumoral injection model to further validate whether the enhanced metastasis was attributed to the DAMPs released by necroptotic cells (Fig. 2K). Results showed that tumors injected with necroptotic DAMPs had a significantly higher metastatic rate than those injected with apoptotic and necrotic DAMPs (Fig. 2L–N and Fig. S1M, N), while there was no significant difference in tumor volume among the groups (Fig. S1O). Collectively, our data suggested that necroptosis played a crucial role in driving tumor-cell invasiveness and lymphatic metastasis via releasing tumor-type specific DAMPs.

### DAMPs-driven reprogramming of peri-necroptotic microenvironment facilitates HNSCC invasiveness

**Fig. 3:**
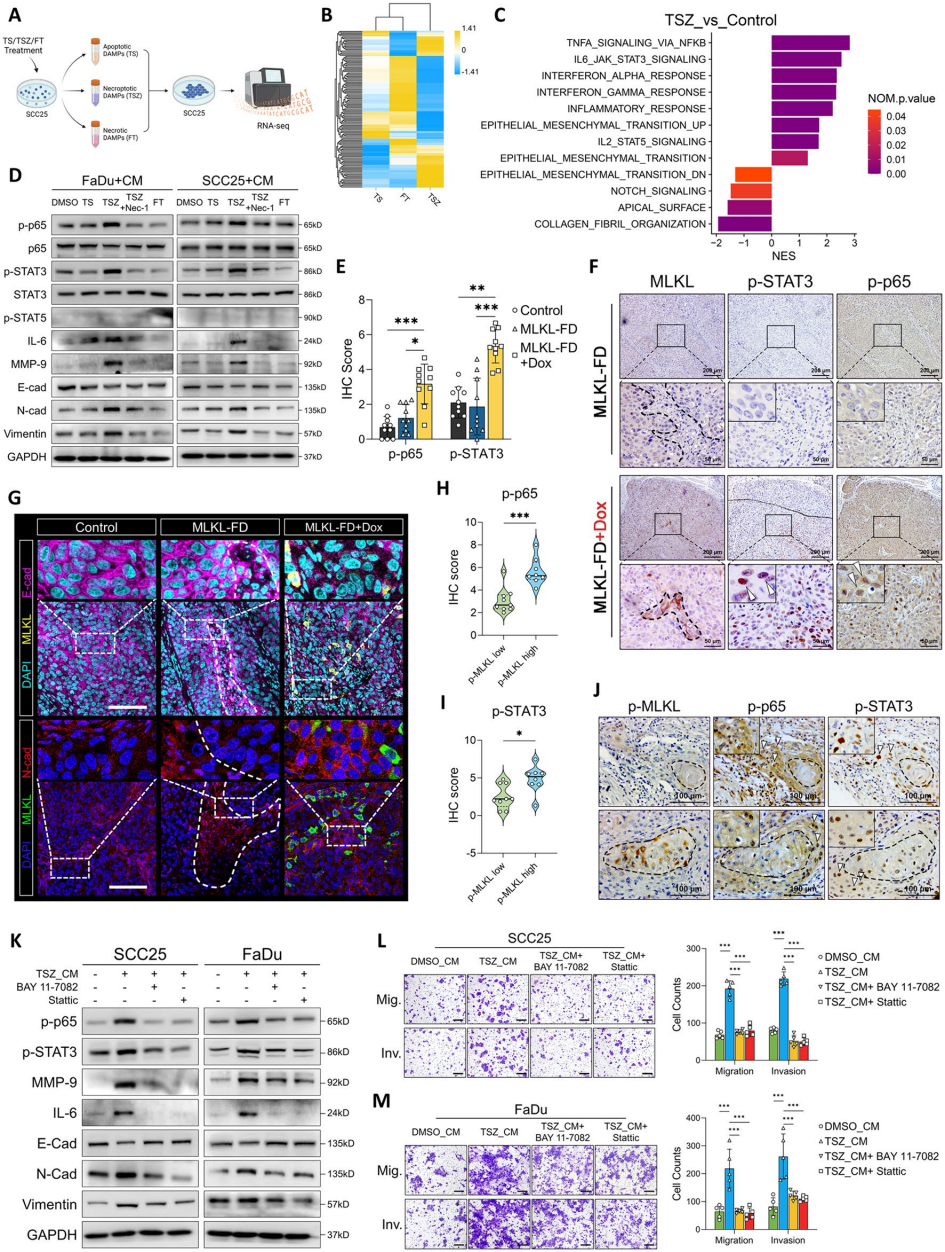
DAMPs-driven reprogramming of peri-necroptotic microenvironment facilitate tumor-cell EMT and invasiveness. A Schematic of the RNA-seq analysis. **B** Hierarchical clustering analysis showing differential expression patterns of SCC25 cells treated by apoptotic (TS), necrotic (FT) and necroptotic (TSZ) DAMPs. **C** GSEA results of signal alterations in TSZ group comparing to Control group (TS + FT). **D** SCC25 and FaDu were treated by different CM for 24 h. The activation of NF-κB, STAT3 and STAT5 signaling and the expression of EMT markers were detected by Western blotting. **E** IHC of p-p65 and p-STAT3 were performed to the xenograft tissue sections from the Dox-induced necroptosis model. The overall IHC score were compared by one-way ANOVA and Turkey’s multiple comparisons test. **F** Representative image of p-p65 and p-STAT3 staining around MLKL^high^ necroptosis in Dox-induced group and MLKL^low^ necrosis in uninduced group. The necrotic and necroptotic clusters were outlined by dotted line, respectively. **G** Multi-immunofluorescent staining was performed to the three groups of xenograft tissue sections from the Dox-induced necroptosis model. Representative image of E-cad and N-cad staining in non-necrotic, necrotic and necroptotic microenvironment were shown. Necrotic clusters in MLKL-FD tumor sections were outlined by dotted line (scale bar = 100μm). **H**–**J** Primary HNSCC tissues were divided into high-necroptosis (p-MLKL high) and low-necroptosis (p-MLKL low) group based on p-MLKL staining. Then the IHC staining of p-p65, and p-STAT3 were conducted on the serial sections of each group. The IHC score was quantified and compared (**H**, **I**). Representative image of p-p65, p-STAT3 activation in peri-necroptotic cells were shown in (J). P-MLKL + necroptotic clusters were outlined by dotted line. **K**–**M** SCC25 and FaDu were pre-treated with BAY 11-7082 or Stattic for 1 h and then treated with necroptotic DAMPs for 24 h. Protein expression and tumor-cell migration and invasion were analyzed by **K** western blotting and **L**, **M** Transwell assays respectively (Scale bar = 200μm, n = 5). *p < 0.05, **p < 0.01, ***p < 0.001

To investigate how necroptotic DAMPs affect peri-necroptotic microenvironment, we used RNA-seq to analyze the transcriptomic alterations in SCC25 cells treated with apoptotic (TS), passive-necrotic (FT) and necroptotic (TSZ) DAMPs (Fig. 3A). Results showed a distinct gene-expression pattern in necroptotic-DAMPs treated cells (Fig. 3B and Fig. S2A). The top 30/20 enriched gene sets from GO/KEGG databases were mainly annotated by level 2 terms including “immune system”, “signal transduction”, “signaling molecules and interactions”, “response to stimulus” and “biological regulation” (Fig. S2B-E). Differential genes were enriched in multiple cytokine- and signaling pathway-related gene sets, such as cytokine-cytokine receptor interaction (ko04060), IL-17 signaling pathway (ko04657), NF-kappa B signaling pathway (ko04064), Jak-STAT signaling pathway (ko04630), etc. (Fig. S2F, G). Further gene-set enrichment analysis (GSEA) confirmed the upregulation in several cytokine-associated signalings including NF-κB, IL6-JAK-STAT3, IL2-STAT5, and IFN-α pathways, as well as epithelial mesenchymal transition (EMT), which is a crucial machinery for tumor-cell dissemination (Fig. 3C). In addition, genes harboring the binding site for STAT3 was also significantly enriched in the TSZ group, further supporting the activation of the JAK-STAT3 pathway (Fig. S2H). Together, these data suggested that necroptotic DAMPs may exert cytokine-like functions and reshape peri-necroptotic microenvironment via activating downstream signalings.

As NF-κB, JAK-STAT3, and IL2-STAT5 were related to tumor progression [34–36], we then tested whether they mediated the DAMPs-driven reprogramming of peri-necroptotic microenvironment. Comparing with apoptotic and passive-necrotic DAMPs, necroptotic DAMPs can induce increased phosphorylation and nuclear translocation of p65 and STAT3, but not STAT5, along with the upregulation of their downstream targets IL-6 and MMP-9 in peri-necroptotic cells, suggesting the activation of NF-κB and STAT3 signaling (Fig. 3D and Fig. S2K). In addition, consistent with the RNA-seq results, a panel of NF-κB- and STAT3-downstream target genes (Fig. S2I) including cytokines/chemokines (*CXCL8, CXCL1, IL6, CXCL2, ILA*) and proliferation-, migration- and invasion-related genes (*PLAU, MMP9, MMP1, ICAM1, CDKN1A, BIRC3*) were upregulated in necroptotic-DAMPs treated cells (Fig. S2J). Moreover, the xenograft tumors with dox-induced intratumoral necroptosis showed significantly enhanced STAT3 and NF-κB activation (Fig. 3E). Importantly, enhanced NF-κB and STAT3 activation were seen in peri-necroptotic (MLKL^high^) microenvironment while absent around site of accidental necrosis (Fig. 3F). Moreover, decreased E-cadherin expression and increased N-cadherin expression were seen in peri-necroptotic tumor cells but not peri-necrotic cells, which, in consistent with GSEA results, suggesting an upregulation of EMT in peri-necroptotic microenvironment (Fig. 3G). Accordingly, necroptotic DAMPs induced the upregulation of the mesenchymal markers N-cadherin and vimentin and invasive marker MMP-9, while the epithelial marker E-cadherin was downregulated (Fig. 3D) in peri-necroptotic cells. Additionally, in HNSCC patients’ samples, primary tumors with extensive necroptosis showed higher level of NF-κB and STAT3 activation (Fig. 3H–J). Either inhibition of NF-κB or STAT3 can attenuate the tumor-cell EMT and invasiveness induced by necroptotic DAMPs (Fig. 3K–M and Fig. S2L). Collectively, we demonstrated that necroptotic DAMPs drive peri-necroptotic EMT and invasiveness through activating NF-κB and STAT3 signalings.

### Proteomic landscape of necroptosis-specific DAMPs in HNSCC

**Fig. 4:**
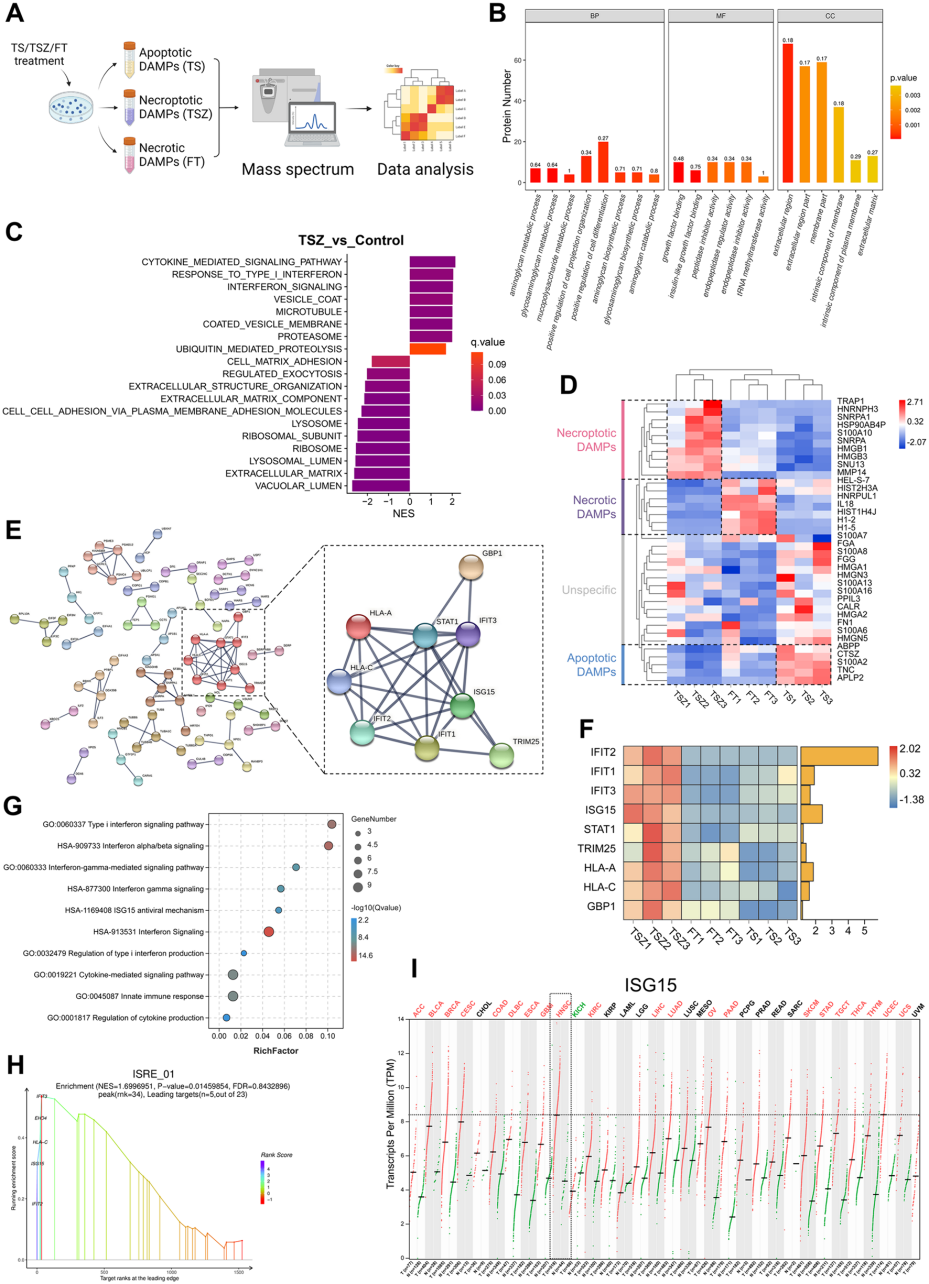
Proteomic landscape of HNSCC-specific necroptotic DAMPs. **A** Schematic of label-free proteomic analysis of apoptotic, necrotic and necroptotic DAMPs in HNSCC. **B** GO enrichment analysis of the differential proteins in necroptotic (TSZ) DAMPs comparing to apoptotic (TS) and necrotic (FT) DAMPs. **C** GSEA results showing compositional and functional alterations in necroptotic DAMPs. **D** Hierarchical clustering of selected DAMPs among three groups. **E** PPI analysis and MCL clustering of upregulated proteins in TSZ group. Members comprising the biggest cluster were shown in the right panel, and their expression in our proteomic assay were shown in (**F**). **G** Enrichment analysis of the functional cluster presented in the right panel of (**E**). **H** GSEA results showing the transcriptional binding site ISRE were enriched in the necroptotic DAMPs. **I** Expression profile of ISG15 across various cancer types were plotted from TCGA database using GEPIA (http://gepia.cancer-pku.cn/index.html)

Our results indicated that instead of facilitating the passive leakage of intracellular components (as DAMPs from FT group failed to induce signal alterations and tumor-cell EMT), necroptotic cells produce functional DAMPs in an active manner, which further drives reprogramming of peri-necroptotic microenvironment. To comprehensively reveal the specific DAMPs composition of necroptosis in HNSCC, we next profiled the proteins released during apoptosis, necrosis, and necroptosis using a label-free proteomic technique (Fig. 4A). As expected, the protein composition of necroptotic DAMPs is significantly different from that of apoptotic and necrotic DAMPs (Fig. S3A). The differentially expressed proteins were mainly located in the cytoplasm, nucleus, and extracellular region (Fig. S3B). Differential proteins were enriched in multiple gene sets related to the extracellular region, as well as the biosynthesis and metabolism of matrix components, including glycosaminoglycans (GAGs), N-glycans and glycoproteins (Fig. 4B and Fig. S3C), which were previously reported to be DAMPs [37], suggesting change in their release. Further GSEA confirmed the decreased release of extracellular matrix components in necroptosis (Fig. 4C). A previous study reported that lysosomal components are released in the early stage of necroptosis [38]. Here, conversely, lysosomal and ribosomal components were downregulated in necroptotic DAMPs (Fig. 4C). Instead, we observed a upregulated proteasomal functions and release of proteasomal components in necroptosis group (Fig. 4C). Since lysosomes and proteasomes play essential roles in proteolysis, their functional modifications likely contribute to the differential DAMP release in necroptosis. In addition, the upregulation of vesicle and microtubule components suggests enhanced intracellular vesicle transportation in necroptotic cells (Fig. 4C), which is consistent with previous studies [39, 40]. Then, we used hierarchical clustering analysis to compare the release of a panel of selected proteins that have been previously reported as DAMPs [37, 41, 42]. Based on the differential expression, the selected proteins were divided into apoptosis-, necrosis- and necroptosis-specific DAMPs (Fig. 4D). Certain patterns of DAMP composition can be seen in different types of cell death. For instance, histone components are specifically upregulated in necrotic DAMPs, whereas multiple ribonucleoproteins and heat shock proteins (HSPs) are exclusively released during necroptosis. The release of the HMG and S100 protein families, which are canonical DAMP components, seems to be nonspecific, as the upregulation of their various members was detected in all three groups (Fig. 4D).

However, the abovementioned canonical DAMPs, such as HMGs, S100s, HSPs, ribonucleoproteins and glycans, are often widely expressed among different tissues and have been reported in the context of multiple types of cell death. Hence, they were unlikely to contribute to necroptosis-related disease progression in HNSCC. Therefore, we next sought to identify HNSCC-specific components in necroptotic DAMPs. We analyzed the functional interactions among proteins upregulated in the TSZ group using the STRING database. Protein–protein interaction (PPI) and MCL clustering analysis retrieved 27 functional clusters; the largest cluster comprised nine proteins: IFIT1, IFIT2, IFIT3, ISG15, STAT1, TRIM25, HLA-A, HLA-C, and GBP1 (Fig. 4E, F). Interestingly, the proteins in this cluster are all members of the interferon-stimulated gene (ISG) family and are enriched in multiple gene sets related to interferon-mediated processes (Fig. 4G). In addition, GSEA showed that the interferon alpha/gamma response and the interferon-sensitive response element (ISRE), which is the key transcriptional binding site for the induction of ISG expression [28], were upregulated in the TSZ group (Fig. 4H and Fig. S3D, E), suggesting that interferon signaling may be involved in the production of these proteins during necroptosis. We then plotted the expression profile of the top four upregulated ISGs (IFIT2, ISG15, IFIT1 and IFIT3) among various cancer types from the TCGA database. Interestingly, these four ISGs, especially IFIT3 and ISG15, are all highly upregulated in HNSCC tissues (Fig. 4I and Fig. S3F-H), indicating that these ISGs may be necroptotic DAMPs with high HNSCC specificity. Together, our data provided a comprehensive landscape of necroptosis-specific DAMPs in HNSCC which was featured by a group of ISGs.

### Programmatic release of ISGs as HNSCC-specific DAMPs via intrinsic orchestration of necroptotic and cGAS-STING signaling

**Fig. 5:**
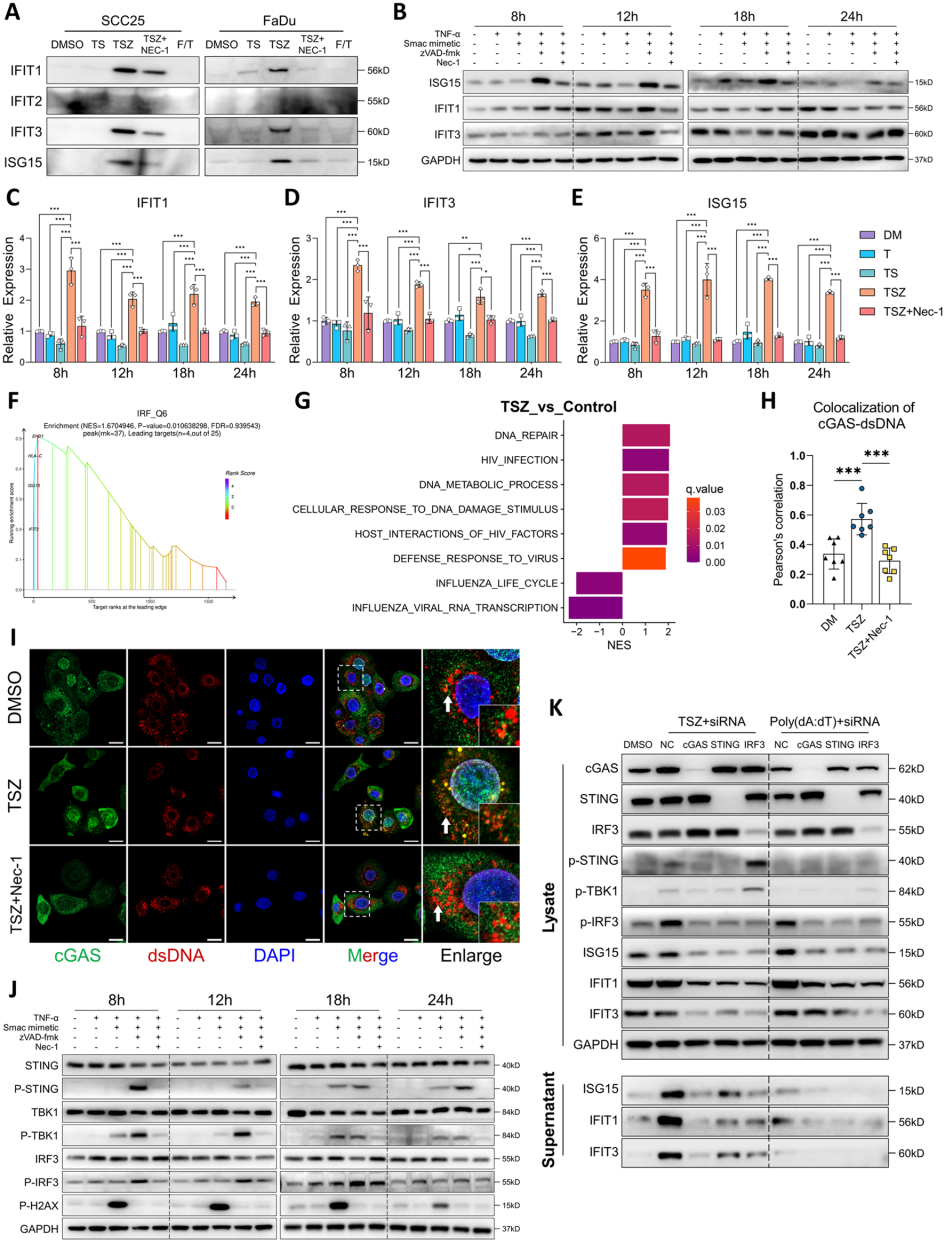
Necroptotic cells actively produce ISGs via intrinsic activation of cGAS-STING. **A** Necroptosis was induced by TSZ. The release of IFIT1, IFIT2, IFIT3 and ISG15 in the supernatants were detected by western blotting. SCC25 cells were treated for 8, 12, 18 and 24 h, the expression of ISG15, IFIT1 and IFIT3 in the cell lysate were analyzed by **B** western blotting and **C**–**E** qRT-PCR. **F** GSEA plot showing necroptotic DAMPs were enriched with transcriptional factor IRF binding sites. **G** GSEA showing alterations in cellular functions. **H**, **I** SCC25 cells were treated by DMSO, TSZ, TSZ + Nec-1 respectively for 8 h, the colocalization of cGAS and dsDNA were analyzed by immunofluorescence (scale bar = 20μm). **J** SCC25 cells were treated for 8, 12, 18 and 24 h, the activation of cGAS-STING-IRF3 signaling and DNA damage were detected by western blotting. **K** SCC25 cells were pre-transfected with siRNA targeting cGAS, STING and IRF3 respectively, cells were then treated with TSZ or Poly(dA:dT) for 12 h. Alterations in protein expression were detected by western blotting. *p < 0.05, **p < 0.01, ***p < 0.001

As the proteomic data indicated that a group of ISGs could be HNSCC-specific necroptotic DAMPs, we further validated this hypothesis. Consistent with the proteomic results, the release of IFIT1, IFIT3 and ISG15 to the supernatant was significantly upregulated in the context of both TSZ- and Dox-induced necroptosis (Fig. 5A and Fig. S4A), which can be inhibited by Nec-1, suggesting their release was cell-death dependent (Fig. 5A). However, the release of IFIT2 was not detected (Fig. 5A and Fig. S4A). In addition to their elevated release, we also observed early upregulation of both the protein and mRNA levels of these ISGs in cell lysates after 8 h of TSZ induction (Fig. 5B–E), supporting that these ISGs were actively produced and released during necroptosis, rather than passive leakage of pre-existing proteins. Next, we attempted to reveal the intracellular mechanism involved in the production of ISGs during necroptosis. Using GSEA, we identified that genes containing the transcription factor binding site of interferon regulatory factor (IRF), which is a canonical ISG inducers, were significantly enriched in necroptotic DAMPs (Fig. 5F). Moreover, functions regarding the cellular response to DNA damage stimuli, DNA repair and defense against viral infections were also upregulated in TSZ group (Fig. 5G). Since the cytosolic DNA sensor cGAS and its downstream STING-IRF signaling are essential machineries that mediate the IFN response during DNA damage and viral infection, we suspected that it may be involved in the ISGs production during necroptosis. Consistently, increased cytoplasmic colocalization of cGAS and dsDNA (Fig. 5H, I) and activation of the STING-TBK1-IRF3 pathway were seen in early necroptosis while they were absent in solely TNF-α treated cells and TS induced apoptosis (Fig. 5J). Increased cGAS-dsDNA colocalization and STING-TBK1-IRF3 phosphorylation were abolished upon inhibition of necroptosis by Nec-1, confirming a necroptotic-dependent activation of cGAS-STING (Fig. 5I, J). Since cGAS-STING is known to mediate type I IFN production [43], we also tested the expression and release of IFNβ. Of note, while the mRNA level of *IFNB* was upregulated in necroptosis (Fig. S4B), its release was not significantly elevated (Fig. S4C). It was not detected in our proteomic assay either. Similar results are reported by Tanzer et al. [38] which report that necroptotic cells showed necroptotic cells showed reduced secretion of conventional cytokines [38]. Since many interferons are known to be produced in a transient manner and are subjected to various post-transcriptional, translational, and post-translational regulations [44–47], our data implied that the restricted release of traditional cytokines in necroptotic cells might be due to post-transcriptional regulation.

Next, to test the relation between cGAS-STING activation and ISGs production, cells were pre-transfected with siRNA targeting cGAS, STING and IRF3 before TSZ treatment. Abolishing cGAS-STING-IRF3 signaling strongly inhibited the expression and release of IFIT1, IFIT3 and ISG15 in necroptotic cells (Fig. 5K and Fig. S4D). Similar results were observed when cGAS and STING were functionally blocked by G150 and C170, respectively (Fig. S4E). Importantly, extrinsic dsDNA mimics poly(dA:dT) can also induce the expression of the tested ISGs via activation of cGAS-STING, yet cells fail to release these ISGs into the extracellular space (Fig. 5K and Fig. S4E), suggesting that necroptotic cell death is an indispensable route for ISGs release.

**Fig. 6:**
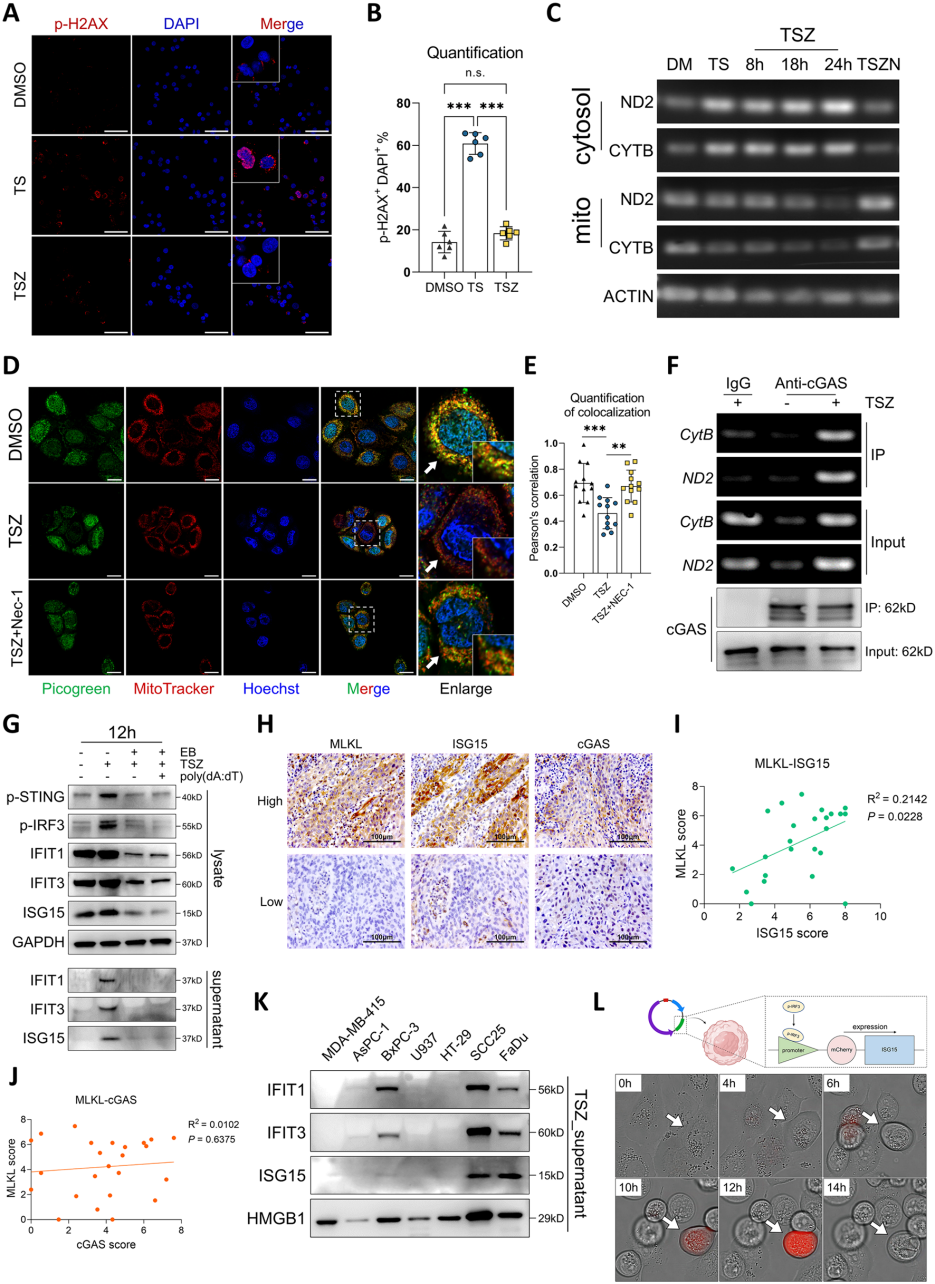
Programmatic release of ISGs through mtDNA-cGAS-IRF3 axis. **A**, **B** SCC25 cells were treated with DMSO, TS and TSZ for 8 h, immunofluorescent staining of p-H2AX was performed to analyze the level of DNA damage (scale bar = 100μm). **C** SCC25 cells were treated by DMSO, TS, TSZ + Nec-1 for 24 h and by TSZ for 8, 18 and 24 h. DNA were extracted from mitochondrial and cytosolic fractions respectively. The cytosolic release of mitochondrial DNA was analyzed by genomic PCR and DNA electrophoresis. **D**, **E** SCC25 cells were pre-stained with Picogreen, MitoTracker and Hoechst 33,342, then treated with DMSO, TSZ and TSZ + Nec-1 for 8 h. The cytosolic mtDNA release was visualized by analyzing the colocalization of Picogreen and MitoTracker (scale bar = 20μm). **F** SCC25 cells were treated by DMSO or TSZ for 8 h, then cytosolic cGAS were pulled down and DNA were extracted from the precipitate. The binding of cGAS and mtDNA was analyzed by genomic PCR and DNA electrophoresis. **G** EB were used to deplete mtDNA in SCC25 cells. Cells were then treated with TSZ with or without poly(dA:dT) for 12 h. The cGAS-STING activation, ISGs expression and release were detected by western blotting. **H**–**J** IHC staining were performed in HNSCC primary tumor sections, the representative images of high- and low- expression of MLKL, ISG15 and cGAS were shown in (**H**, scale bar = 100μm). The correlation between MLKL-ISG15 and MLKL-cGAS expression was analyzed respectively (**I**, **J**). **K** Cells with various histologic origins were treated by TSZ for 24 h, the release of IFIT1, IFIT3, ISG15 and HMGB1 in the supernatant were detected by western blotting. **L** SCC25 stably transfected with ISG15 promoter-mCherry-ISG15 lentivirus were treated by TSZ. Changes in cellular morphologies and mCherry fluorescent signals were continuously observed by PE Operetta CLS (PerkinElmer, USA) imaging system. n.s. not significant, *p < 0.05, **p < 0.01, ***p < 0.001

However, the activation of cGAS in necroptosis is unlikely to be triggered by canonical DNA damage, as necroptotic cells did not show upregulated level of p-H2AX (Figs. 5J and 6A, B). Since necroptosis is executed through the pore-forming ability of p-MLKL, we suspected the dsDNA that triggered cGAS-STING signaling may be derived from mitochondria whose membrane integrity is disrupted in early necroptosis. To test this hypothesis, we performed PicoGreen-MitoTracker double staining and genomic PCR, both of which showed a dramatic release of mtDNA into the cytosol in the early stage of necroptosis (Fig. 6C–E). Continuous live imaging confirmed that the release of mtDNA was an early event during necroptosis (up to 6 h after TSZ induction), whereas morphological changes and cell lysis occurred in the later stage (Fig. S4F). Inhibition of necroptosis significantly rescued the cytosolic release of mtDNA, supporting a necroptosis-driven mitochondrial permeabilization (Fig. 6C–E). Immunoprecipitation of cGAS followed by genomic PCR further validated the enhanced cGAS-mtDNA binding during necroptosis (Fig. 6F). Next, to confirm that the STING activation and ISGs production were induced by cytosolically released mtDNA, we used ethidium bromide (EB) to deplete the mtDNA before inducing necroptosis (Fig. S4G). As results, the activation of cGAS-STING signaling, ISGs expression and release in necroptosis were all dramatically abolished (Fig. 6G and Fig. S4H). Together, we demonstrated the programmatic production and release of ISGs via mtDNA-cGAS-IRF3 axis during necroptosis. Interestingly, the addition of poly(dA:dT) failed to restore the ISGs release in mtDNA-depleted cells (Fig. 6G and Fig. S4H), which may indicate an impaired uptake of exogeneous DNA in necroptotic cells. This in turn supports a necroptotic cell-intrinsic machinery for ISG induction and release.

Next, we explored the relation between necroptosis and interferon signaling in HNSCC patients using TCGA database. By analyzing *MLKL*-co-expressed genes, we found that the expression of ISGs identified in Fig. 4E as well as *CGAS* were all correlated with *MLKL* expression in HNSCC patients (Fig. S4I-O and Fig. S5A-C). The expression of *BAK1*, which is a key regulator of mitochondrial membrane permeability [48], was also correlated with *MLKL* expression (Fig. S5D). Further validation using clinical specimens confirmed a significant co-expression of ISG15 with MLKL, while no significant correlation was observed between cGAS and MLKL expression (Fig. 6H–J). Next, we queried the top 200 *MLKL*-co-expressed genes in STRING database. The PPI analysis and MCL clustering retrieved a major cluster which comprised of 49 genes (Fig. S5E, F) and were functionally enriched in multiple gene sets regarding interferon signaling and defense against viral infections (Fig. S5G). Together, these results suggested an intrinsic correlation between necroptosis and interferon signaling mediated by cellular nucleic-acid sensors in HNSCC.

As we hypothesized that these ISGs may be HNSCC-specific necroptotic DAMPs, we then compared the necroptotic release of these ISGs in various cell lines derived from breast, pancreatic, colorectal cancer, and histiocytic lymphoma. As results, the necroptotic release of ISGs were absent in these cells except for BxPC-3 cells showing mild release of IFIT1 and IFIT3 (Fig. 6K). TGF-β, VEGFA were known to promote tumor-cell EMT and metastasis, respectively, and secreted cathepsins such as Cathepsin L and Cathepsin B were reported to facilitate tumor-cell invasion. However, apart from a slightly higher release of Cathepsin L in SCC25 compared to U937, there was no significant difference in the release of these molecules during necroptosis among different cancer types (Fig. S5H-K). Together, our data supported that these ISGs, especially for ISG15, were HNSCC-specific necroptotic DAMPs.

Finally, we transfected SCC25 cells with a lentiviral vector containing an ISG15 promoter (including ISRE) followed by an mCherry-ISG15 fused gene, so the expression of the mCherry-ISG15 fusion protein could only be induced upon the appearance of ISRE-binding transcription factors (Fig. 6L). Through continuous live imaging, we visualized the programmatic production of ISG15 during necroptosis: the fluorescent mCherry-ISG15 proteins were actively induced in early necroptotic cells with the appearance of necrotic morphologies (swelling and disconnection) and were quickly released upon rupture of the cellular membrane (Fig. 6L, and Supplementary video 1, 2).

Collectively, we here revealed a well-orchestrated production of ISGs during necroptosis: the cytosolic release of mtDNA driven by necroptosis activates cGAS-STING to induce the expression of ISGs in early stage; the induced ISGs were later released as HNSCC specific DAMPs upon cell death. Our data indicated a cell-intrinsic necroptotic-inflammatory signal orchestration which drives specific DAMPs production during necroptosis.

### Necroptotic ISG15 drives microenvironment reprogramming and lymphatic metastasis via RAGE

**Fig. 7:**
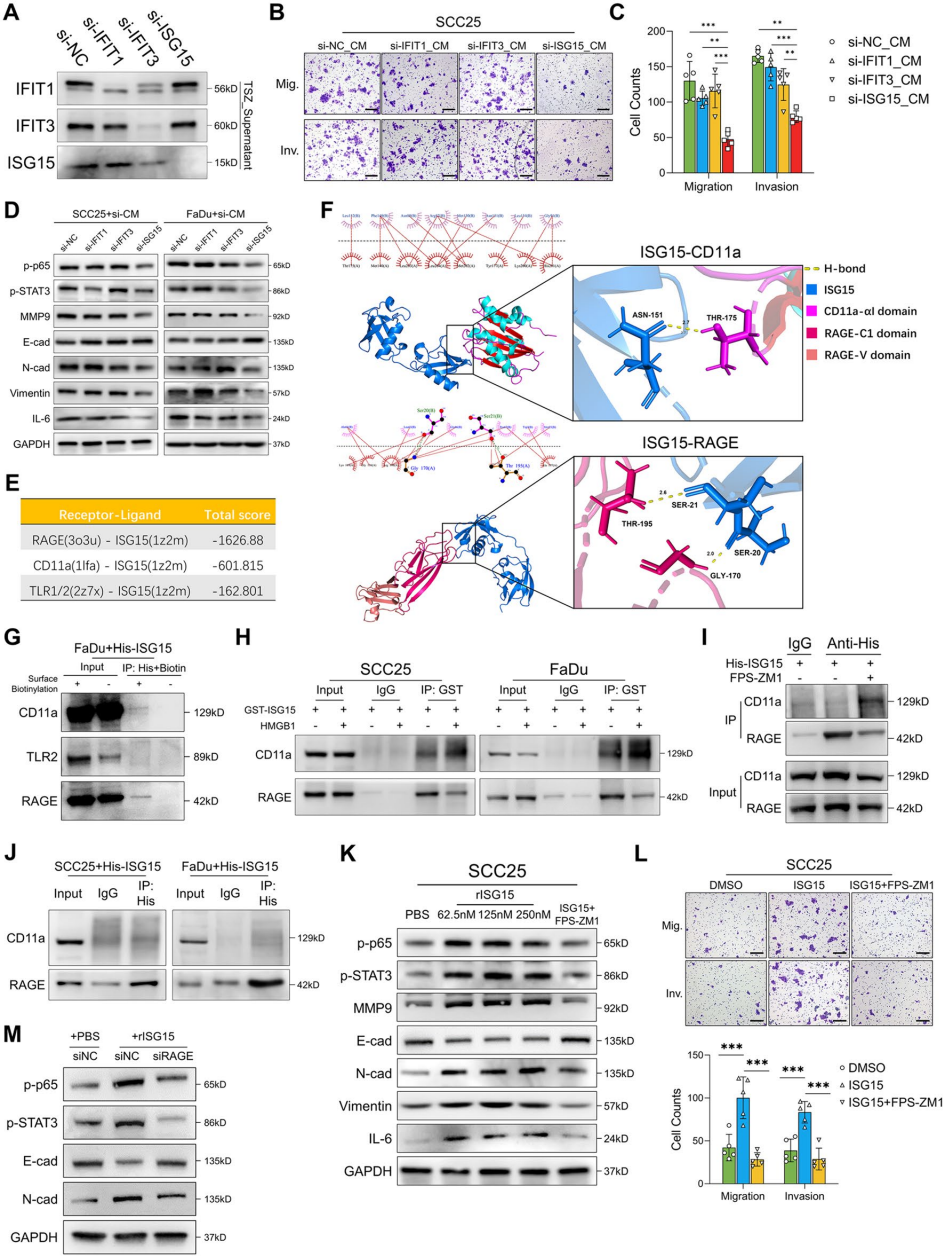
RAGE is a novel receptor for extracellular ISG15. **A** SCC25 cells were pre-transfected with siRNA targeting IFIT1, IFIT3 and ISG15 then treated with TSZ for 24 h. The release of IFIT1, IFIT3 and ISG15 in the supernatants were detected by western blotting. **B**, **C** SCC25 cells were treated by IFIT1/IFIT3/ISG15-depleted necroptotic DAMPs for 24 h. Transwell assays were used to analyze tumor-cell migration and invasion (scale bar = 200 μm, n = 5). **D** SCC25 and FaDu cells were treated by IFIT1/IFIT3/ISG15-depleted necroptotic DAMPs for 24 h. Protein-level alterations were detected by western blotting. **E** The total docking score of RAGE-ISG15, CD11a-ISG15, and TLR1/2-ISG15. **F** Visualization of the binding conformations of ISG15-RAEG and ISG15-CD11a with the lowest docking score. **G** FaDu cells with or without surface-protein biotinylated were treated by His-ISG15 for 4h. Sequential IP of His and Biotin were performed and then the CD11a, TLR2 and RAGE in the precipitates were detected by western blotting. **H** SCC25 and FaDu cells were treated with recombinant GST-ISG15 and HMGB1 or GST-ISG15 alone for 2 h. Recombinant GST-ISG15 were then pulled down by IP and its binding with RAGE and CD11a were detected by western blotting. **I** SCC25 cells were pre-treated by 10uM FPS-ZM1 for 1 h followed by His-ISG15 treatment for 2 h. His-ISG15 were then pulled down by IP and its binding with CD11a and RAGE were analyzed by western blotting. **J** SCC25 and FaDu cells were treated with recombinant His-ISG15 for 2 h. His-ISG15 were then pulled down by IP and its binding with RAGE and CD11a were detected by western blotting. **K**, **L** SCC25 cells were treated by recombinant ISG15 (rISG15) or rISG15 + FPS-ZM1 for 24 h, western blotting was performed to analyze downstream protein alterations (**K**) and tumor-cell migration and invasion were analyzed by Transwell assays (**L**, scale bar = 200 μm, n = 5). **M** FaDu cells transfected with siRNA targeting RAGE (siRAGE) or negative control (siNC) were treated by 250nM rISG15 for 24 h. Downstream protein alterations were analyzed by Western blotting. *p < 0.05, **p < 0.01, ***p < 0.001

Since we demonstrated that IFIT1, IFIT3 and ISG15 are programmatically released as HNSCC-specific DAMPs during necroptosis, we then tested whether they contributed to the DAMPs-driven invasiveness in peri-necroptotic cells. We knockdown the expression of IFIT1/3 and ISG15 in HNSCC cells (Fig. S6A-C), which abolished their release during necroptosis (Fig. 7A). We noticed that depletion of necroptotic ISG15 significantly abolished the DAMPs-driven invasiveness in vitro (Fig. 7B, C and Fig. S6D, E). Moreover, ISG15-depleted necroptotic DAMPs failed to induce NF-κB and STAT3 activation and tumor-cell EMT (Fig. 7D). Together, these data suggested a crucial function of ISG15 in necroptotic DAMPs. As we showed that necroptotic DAMPs exert cytokine-like functions to reshape peri-necroptotic microenvironment, we next sought to identify the surface receptor for extracellular ISG15. A previous study reported that extracellular ISG15 can bind to the integrin receptor LFA-1 (CD11a) on NK cells and activate downstream SRC family kinases (SFKs) [49]. However, the PI3K-Akt and MAPK signaling pathways, which are canonical downstream signaling pathways of SFKs [50], were not enriched according to GSEA (Fig. S6F, G), suggesting that ISG15 may function through alternative receptors. To determine the alternative receptors for extracellular ISG15, we selected two PRRs, TLR1/2 and RAGE, which are known to activate downstream NF-κB and STAT3 signals [37] and utilized molecular docking to simulate their receptor–ligand binding with ISG15. Results showed an unlikely ligand–receptor interaction between ISG15 and TLR1/2, indicated by a high docking score (Fig. 7E). However, the docking score of ISG15-RAGE was significantly lower than that of the positive control ISG15-CD11a (Fig. 7E). Further analysis showed that the N-terminus of ISG15 binds to the C1 ectodomain of RAGE through two H-bonds (S20-G170 and S21-T195) and multiple hydrophobic bonds, whereas the C-terminus of ISG15 and the αI domain of CD11a form only one H-bond with a longer bond length (Fig. 7F), indicating that RAGE is not only a potential receptor for extracellular ISG15 but also has a higher affinity for ISG15 than CD11a. To validate our hypothesis, we used coimmunoprecipitation (co-IP) to detect whether extracellular ISG15 can bind to RAGE. The results showed that extracellular ISG15 can bind to both CD11a and RAGE, while the positive control HMGB1 only binds to RAGE (Fig. S6H). Next, we used biotin to label the cell-surface protein and then treated cells with His-tagged ISG15. Sequential immunoprecipitation of His and Biotin confirmed that the ISG15 binds to the surface RAGE as well as the positive control CD11a, but not TLR2 (Fig. 7G). Moreover, the binding of ISG15 and RAGE can be either competitively reduced upon the addition of HMGB1 (Fig. 7H) or inhibited by RAGE inhibitor FPS-ZM1 (Fig. 7I), suggesting that ISG15 binds to RAGE in a ligand–receptor manner. Furthermore, His-ISG15 was used to treat HNSCC cells and then pulled down by immunoprecipitation. Western blotting (WB) showed significant enrichment of RAGE in the precipitate, while the CD11a level was lower comparing to the input (Fig. 7J), suggesting that ISG15 preferentially binds to RAGE. Similar results were observed when the targets for IP and WB were switched in the same system (CD11a and RAGE were pulled down, respectively, and His-ISG15 was detected by WB, Fig. S6I). In addition, increased ISG15-CD11a binding were seen when binding between ISG15 and RAGE were competitively reduced by HMGB1 (Fig. 7H) or inhibited by FPS-ZM1(Fig. 7I). Together, our data demonstrated that RAGE is a receptor for ISG15 with higher affinity than CD11a.

**Fig. 8:**
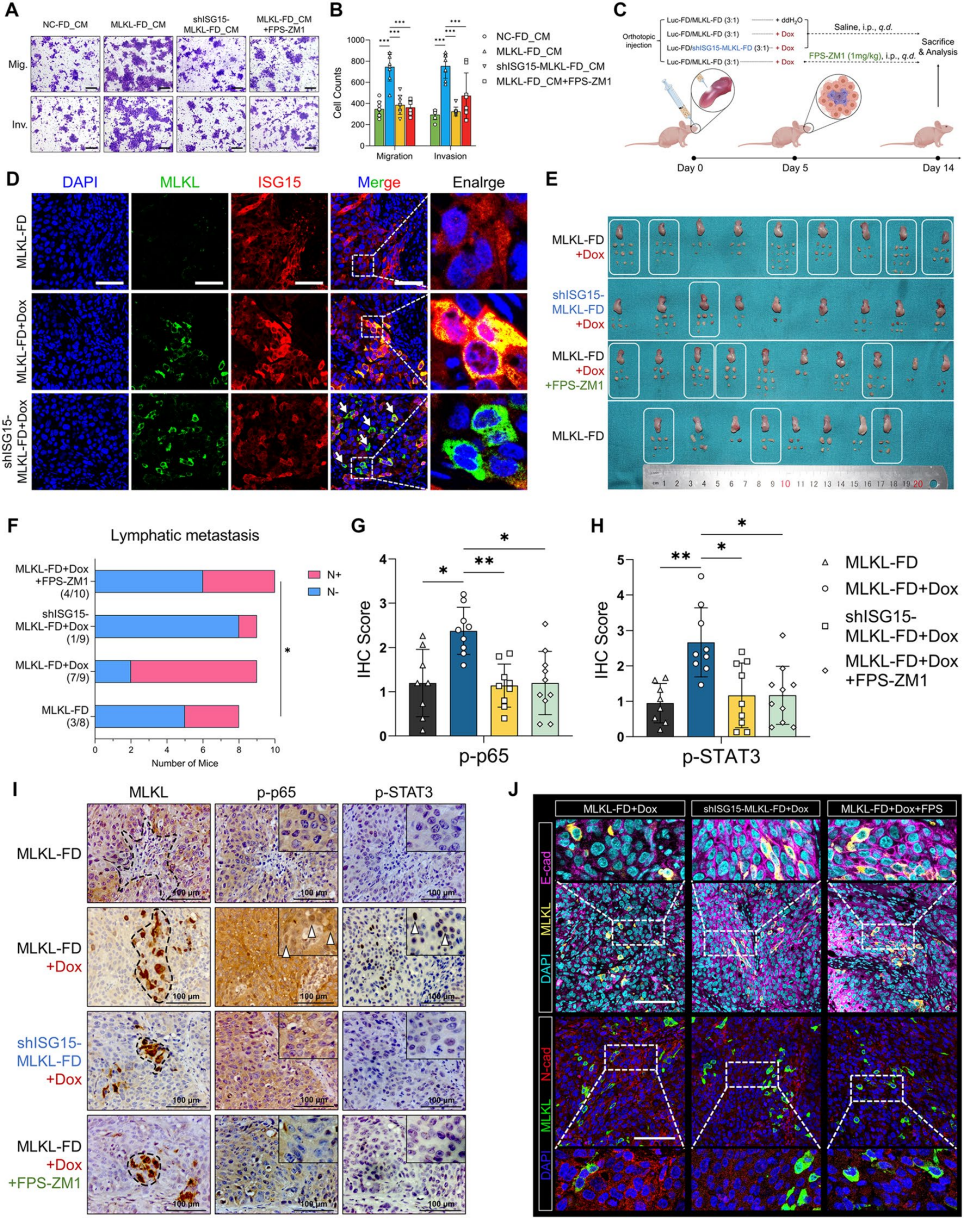
Necroptosis-dependent ISG15 drives lymphatic metastasis via RAGE signaling. **A**, **B** Dox-induce necroptotic DAMPs were collected from MLKL-FD and shISG15-MLKL-FD cells. FaDu cells were treated with NC-FD_CM, MLKL-FD_CM, shISG15-MLKL-FD_CM and MLKL-FD_CM + FPS-ZM1 for 24 h. Tumor-cell migration and invasion were analyzed by Transwell assays (scale bar = 200 μm, n = 8). **C** Schematic of the in vivo assay. **D** Multi-immunofluorescent staining showing ISG15 was knockdown in MLKL^high^ necroptotic cells (scale bar = 60 μm). **E** Tongue and cervical lymph nodes collected at the endpoints. White frame indicated the mice with pathologically validated cervical lymphatic metastasis. **F** Comparison of the cervical metastatic rates among four groups (Fisher’s exact test). **G**, **H** Comparison of the level of NF-κB and STAT3 staining in the primary tumors among four groups. **I** Representative images of MLKL, p-p65 and p-STAT3 staining on serial sections of the xenograft tumors. **J** Representative images showing E-cad and N-cad alterations in peri-necroptotic microenvironment in the xenograft tumors (scale bar = 100 μm). *p < 0.05, **p < 0.01, ***p < 0.001

Next, we assessed whether RAGE mediated the extracellular ISG15-driven peri-necroptotic EMT and invasiveness. Treatment with recombinant human ISG15 induced NF-κB and STAT3 activation, tumor-cell EMT, and promoted tumor-cell migration and invasion in vitro (Fig. 7K, L and Fig. S6J-L). ISG15 activate NF-κB and STAT3 in a dose-dependent manner (Fig. S6M). The pathway activation and tumor-cell EMT induced by ISG15 were blocked by either knockdown of RAGE expression (Fig. S6N) or pharmacological inhibition of RAGE function (Fig. 7K–M and Fig. S6J-L). In addition, either ISG15 knockdown in necroptotic cells or RAGE inhibition abolished the invasive phenotype induced by necroptotic DAMPs (Fig. 8A, B, and Fig. S6O). Finally, we adopted the Dox-inducible intratumoral necroptosis model to test whether necroptotic ISG15 drives microenvironment reprogramming and lymphatic metastasis via RAGE (Fig. 8C). The necroptotic release of ISG15 in MLKL-FD cells were knockdown by shRNA and validated by immunofluorescent staining showing low ISG15 expression in Dox-induced necroptotic cells (Fig. 8D). Consistent with in vitro findings, both RAGE inhibition and necroptotic-ISG15 knockdown significantly attenuated necroptosis-driven lymphatic metastasis (Fig. 8E, F), as well as the overall levels of NF-κB and STAT3 activation in primary tumors (Fig. 8G, H). More importantly, the NF-κB and STAT3 activation and tumor-cell EMT in peri-necroptotic microenvironment were all significantly abolished upon necroptotic-ISG15 knockdown or RAGE inhibition (Fig. 8I, J). Collectively, we here identified RAGE as a novel receptor for extracellular ISG15. And ISG15-RAGE signaling played an essential role in necroptosis-driven microenvironment reprogramming and lymphatic metastasis in HNSCC.

### Necroptosis and RAGE expression correlates with poor prognosis in HNSCC

**Fig. 9:**
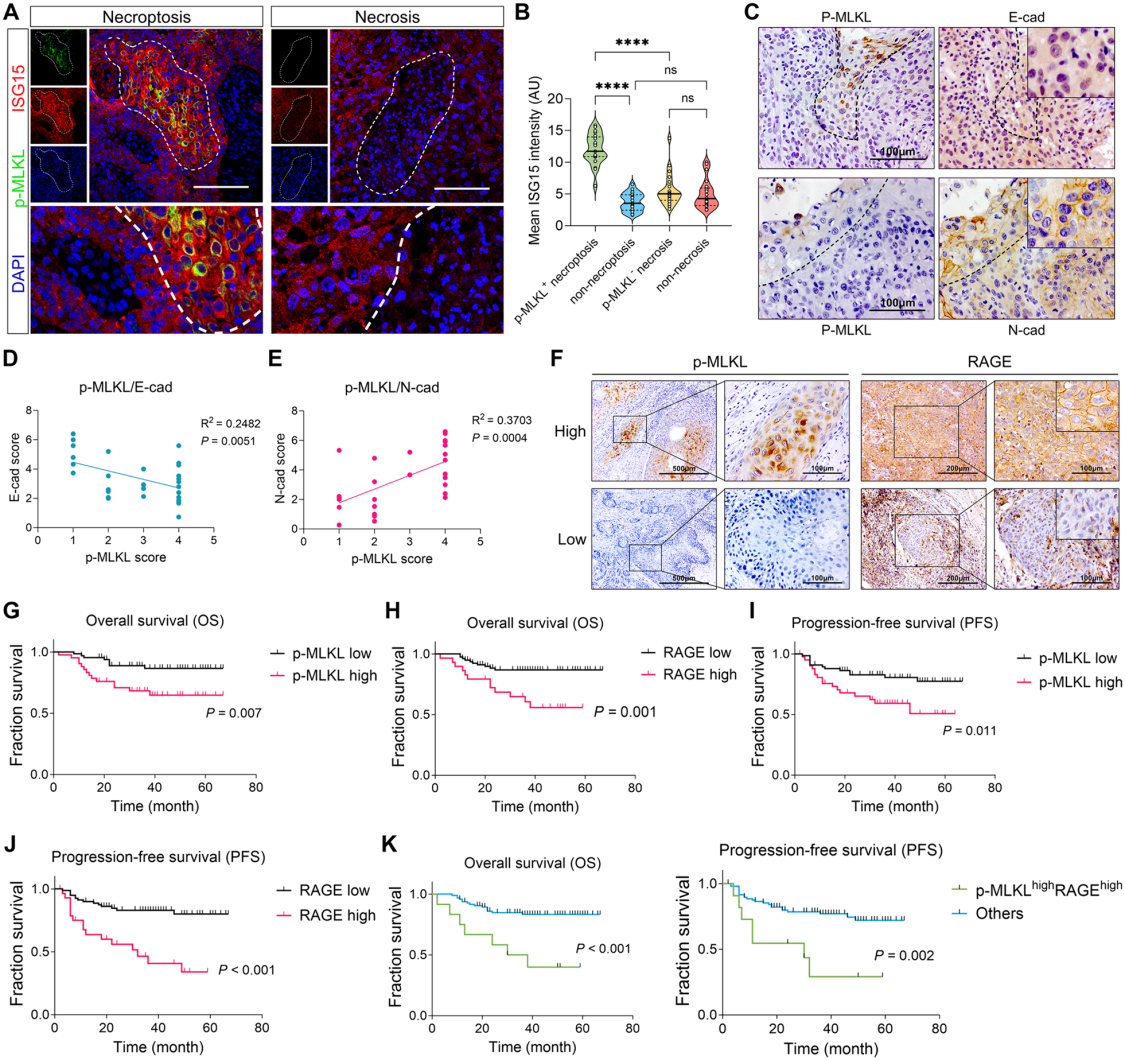
Clinicopathological relevance of necroptosis and RAGE. **A**, **B** Multi-immunofluorescent staining of p-MLKL and ISG15 in HNSCC tumor sections (N = 21). **A** Representative image of p-MLKL^+^ necroptotic foci and p-MLKL^−^ necrotic foci (outlined by dotted line, scale bar = 100μm). **B** Quantification and comparison of ISG15 intensity in indicated area. **C**–**E** IHC staining of p-MLKL, N-cad, E-cad were performed in serial sections of HNSCC patients’ tissue (N = 30). Representative image of peri-necroptotic (p-MLKL^+^, outlined by dotted line) N-cad and E-cad staining were shown in (**C**). The expression of E-cad and N-cad were quantified and their correlation with p-MLKL level were analyzed (**D**, **E**). **F** Representative image of high- and low-expression of p-MLKL and RAGE in HNSCC cells. **G**–**J** Kaplan–Meier survival analysis of the overall survival rate (OS) and progression-free survival rate (PFS) in the p-MLKL high/low and RAGE high/low groups, respectively. The log-rank test was used to compare the survival rate between two groups. **K** Kaplan–Meier survival analysis and log-rank test comparing the OS and PFS between p-MLKL^high^RAGE^high^ patients and other patients. *p < 0.05, **p < 0.01, ***p < 0.001

At last, we sought to test our findings in the clinical setting. Consistent with the in vitro finding, multiplex immunofluorescence staining revealed that the ISG15 signal within p-MLKL + necroptotic foci was significantly higher compared to p-MLKL- necrotic foci and non-necroptotic/necrotic cells (Fig. 9A, B), suggesting a high-ISG15 necroptotic microenvironment in HNSCC. Next, IHC staining in serial sections of HNSCC patients showed that p-MLKL level was negatively correlated with tumor-cell E-cad expression while positively correlated with tumor-cell N-cad expression (Fig. 9C–E), suggesting that extensive necroptosis contributed to HNSCC-cell EMT.

As we demonstrated that necroptotic ISG15 drives HNSCC progression via RAGE signaling, the clinicopathological impact of necroptosis-specific RAGE expression in HNSCC was further investigate. We adopted a 108-patients cohort and analyzed the level of p-MLKL and RAGE expression by immunohistochemical staining (Fig. 9F). As results, high level of p-MLKL or RAGE was not associated with post-treatment recurrence, while both of which significantly correlated with lymphatic metastasis and tumor progression (Table 1). Kaplan–Meier survival analysis showed that extensive necroptosis (p-MLKL high) or high tumor-cell RAGE expression correlated with shorter overall survival rate (OS) and progression-free survival rate (PFS) (Fig. 9G–J). Furthermore, patients with both extensive necroptosis and high tumor-cell RAGE expression (p-MLKL^high^RAGE^high^) showed significant shorter OS and PFS comparing with others (Fig. 9K and Fig. S6P, Q). Taken together, our data suggests that necroptosis-specific ISG15-RAGE axis may contributes to HNSCC progression through promoting tumor-cell dissemination, thus leading to poor prognosis.

**Table 1:** Patients’ clinicopathological characteristics and their correlation with p-MLKL and RAGE expression

Characteristics	RAGE expression				p-MLKL expression		
	N = 108	Low	High	P*	Absent/Focal	Extensive	P*
		n = 79	n = 29		n = 66	n = 42	
Gender							
Male	73	54	19	0.780	40	33	0.052
Female	35	25	10		26	9	
Age							
< 60	80	60	20	0.463	51	29	0.342
≥ 60	28	19	9		15	13	
Tumor location							
Tongue	62	44	18	0.964	40	22	0.098
Buccal	21	16	5		14	7	
Floor of mouth	8	7	1		4	4	
Gingiva	8	6	2		3	5	
Palate	3	2	1		1	2	
Oropharynx	3	2	1		2	1	
Others	3	2	1		2	1	
Tobacco							
No	60	41	19	0.207	40	20	0.185
Yes	48	38	10		26	22	
Alcohol							
No	84	59	25	0.202	53	31	0.429
Yes	24	20	4		13	11	
Areca-nut							
No	98	70	28	0.375	59	39	0.545
Yes	10	9	1		7	3	
T stage							
Tis/1/2	56	42	14	0.652	39	17	0.059
3/4	52	37	15		27	25	
N stage							
N0	58	42	16	0.853	36	22	0.826
N +	50	37	13		30	20	
Clinical stage							
0/I/II	36	27	9	0.759	26	10	0.094
III/IV	72	52	20		40	32	
Histologic grade							
Gx/G0	66	49	17	0.748	37	29	0.177
G1/G2	42	30	12		29	13	
Recurrence							
No	97	71	26	0.973	62	35	0.147
Yes	11	8	3		4	7	
Lymphatic metastasis							
No	88	72	16	** < 0.001**	58	30	**0.032**
Yes	20	7	13		8	12	
Tumor progression							
No	78	65	13	** < 0.001**	53	25	**0.019**
Yes	30	14	16		13	17	

^*^, Chi-square test,* P* < 0.05 was considered statistically significant

## Discussion

Necroptosis was referred as a “double-edged sword” in cancer as both its anti-tumorigenic and pro-tumorigenic role in cancers had been reported [51]. However, such seemingly paradoxical findings are likely to be the consequences of cancers engaging different strategies to overcome survival stress. While some cancers evolve necroptotic resistance to evade stress-induced necroptosis and subsequent immune responses [13, 52], others develop machineries by which exploit necroptosis to fuel their progression [24, 53]. How tumors decide which strategy to adopt is unclear, but it was seemingly related to the tissue origin [5]. For instance, a variety of cancers displayed low/loss expression of RIP3/MLKL and necroptotic resistance [12, 13, 54], while others including pancreatic cancer [55], colorectal cancer [56] and head and neck caner [24] showed high necroptotic rates [12, 13]. In addition, mechanisms by which cancers cope with necroptotic stress are also tumor-type specific. As we showed here that necroptotic cells originated from colorectal, pancreatic cancer and histiocytic lymphoma were incapable of producing ISG15 and inducing invasiveness in HNSCC cells. Instead, colorectal cancer utilizes necroptosis-driven chronic inflammation to foster its tumorigenesis [56–59]. Necroptotic pancreatic cancer cells induce peri-necroptotic immune suppression and invasion through CXCL1, CXCL5 and SAP130-Mincle signalling [55, 60]. In addition, necroptosis microenvironment directs the lineage commitment during liver tumorigenesis via DAMPs-mediated epigenetic reprogramming and thus determining the subtype of liver cancer [61]. Since cancers with diverse histologic origins harbor enormous heterogeneities, future investigations on how cancers “hijack” necroptosis to foster their tumorigenesis and metastasis should fully consider such tumor-type specific factors [1]. Moreover, instead of inhibiting necroptosis, which is currently testing as a potential therapeutic strategy [62], targeting these tumor-type specific machineries may be a more favorable approach to restore the necroptosis-mediated survival stress and thus restrict tumor progression.

Necroptosis affects disease progressions mainly through DAMPs-mediated microenvironment reprogramming [3, 63]. Although much is known regarding necroptotic pathway itself, mechanisms on how necroptosis produce specific DAMPs remain largely obscure [62]. A previous study compared apoptotic and necroptotic DAMPs derived from histiocytic lymphoma cells U937 and human primary macrophages, respectively. As a result, apoptotic cells release more nucleosome components whereas necroptotic cells showed enhanced release of lysosomal components through lysosomal exocytosis, suggesting a differential intracellular regulation during necroptosis [38]. Inconsistently, although we observed similar upregulation of several conventional DAMPs (e.g., HMGB1, HMGB3 and SNRPA1, etc.), our data showed decreased release of lysosomal and ribosomal components and increased proteasome function in necroptotic HNSCC cells. Since the proteasome is the principal machinery for intracellular protein degradation, it may contribute to the differential DAMP release during necroptosis in HNSCC. Importantly, in contrast to our findings, Tanzer’s study showed a significantly decreased ISG15 release and a total absence of IFIT1/2/3 release in necroptosis [38]. This again supported a tissue/disease-specific composition of necroptotic DAMPs which further results in the functional divergence of necroptosis among different cancers.

Emerging evidence have suggested that necroptotic cells produce specific DAMPs in a more programmed manner [38, 64–66]. Instead of passive leakage of cellular components (traditional defined DAMPs), dying cells can still actively shape the microenvironment under the regulation of various intrinsic signals. Here we showed that the cytosolic release of mtDNA is an early event during necroptosis which further activated cGAS-STING intrinsically. Yet the mechanisms regarding mtDNA release are unclear. A previous report suggested that mitochondrial membrane permeabilization in apoptosis and necroptosis may be derived from the mitochondrial outer membrane permeabilization (MOMP) and mitochondrial permeability transition (MPT), respectively [67]. Conversely, transcriptionally induced PUMA was shown to cause the cytosolic release of mtDNA by mediating MOMP during necroptosis [68]. Moreover, necroptosis-activated MLKL can translocate to the mitochondria and induce both MOMP and MPT in a Bax/Bak-dependent manner [69]. Nevertheless, mechanisms on mitochondrial permeabilization during necroptosis still need further inquiry. Caspase-mediated cleavage of cGAS, IRF3 and MAVS have been demonstrated to prevent the activation of cGAS-STING by cytosolic mtDNA released during apoptosis [70–72]. Here, consistently, the cytosolic release of mtDNA was seen in both necroptotic and apoptotic cells, yet cGAS-STING activation and ISG expression were only seen in the context of necroptosis. Our data demonstrated a well-orchestrated procedure of necroptotic HNSCC cells in which specific ISGs expression are induced via mtDNA-cGAS-STING axis at an early stage and later released upon cell death. Similarly, necroptotic cells were reported to autonomously express inflammatory cytokines via RIP1 and RIP3 mediated activation of NF-κB and p38 [73], and the maintenance of endoplasmic reticulum (ER) function holds the mRNA translation even after membrane integrity is disrupted [74]. Collectively, these findings indicated a necroptotic-inflammatory signal orchestration during necroptosis which drives specific DAMPs production, hence providing novel insights into understanding the necroptosis-related inflammation and its unique impact during disease progressions.

In addition to its classical ubiquitin-like protein modification function (ISGylation), ISG15 can also be secreted extracellularly by various cells under the stimulation of type I interferon. Previous studies have reported the cytokine-like functions of extracellular ISG15 including inducing NK cell proliferation, stimulating IFNγ production, and recruiting neutrophils [75–78]. However, the secretory mechanism of ISG15 remains unclear, as it is not secreted by either the canonical Golgi complex or noncanonical multidrug resistance glycoprotein (P-glycoprotein) [29]. Recent studies suggested that extracellular vesicles (EVs) and neutrophil granules are potential routes for the secretion of extracellular ISG15 [79, 80], and its secretion is also regulated by intracellular ISGylation [81]. Interestingly, ISG15 shares multiple similarities with another conventional cytokine, IL-1β, both of which are synthesized from larger precursors, lack secretory signal peptides, and are not secreted via canonical pathways [29]. While IL-1β is a classic cytokine, its secretory mechanism was ambiguous until recent studies demonstrated that in addition to cleaving pro-IL-1β to form mature IL-1β, activated caspase-1 can simultaneously induce pyroptosis through cleavage of Gasdermin D (GSDMD), which in turn allows the release of IL-1β [82]. Herein, similarly, we demonstrated a novel necroptosis-dependent route for ISG15 production and secretion.

Although extracellular ISG15 is known to have cytokine-like functions, its receptor was unclear. The LFA-1 on NK cells was recently demonstrated as a receptor for ISG15 [49]. ISG15 promotes the secretion of IFNγ and IL-10 in IL-12-activated NK cells by binding to the αI domain of CD11a. Therefore, we used CD11a as a positive control in the present study. Through molecular docking and experimental validation, we demonstrated RAGE as a novel receptor for extracellular ISG15 with higher affinity than CD11a. Interestingly, ISG15 was shown to bind to the αI domain of CD11a through its C-terminus [49], and our docking results showed a similar pattern. However, the ISG15-RAGE binding conformation with the highest score showed a N-terminal binding site of ISG15, suggesting that ISG15 may have various binding sites for multiple receptors. RAGE is known to activate downstream NF-κB and STAT3 [37]. However, here we found that ISG15-RAGE-driven activation of NF-κB and STAT3 promotes the invasiveness and metastasis of peri-necroptotic cells, but not cell proliferation and primary tumor growth. This consistent with our previous report as well as study by Cai et al. [24, 83]. Indeed, numerous studies have revealed the role of NF-κB and STAT3 signaling in promoting tumor-cell proliferation, survival, migration, invasion, autophagy, angiogenesis and metastasis in various cancers [84, 85]. However, these effects are not necessarily parallel. In fact, the outcomes of NF-κB and STAT3 activation can be highly context-dependent, not only because they are heterogeneously dysregulated in cancers, but also due to their complex crosstalk with various signalings [84, 85]. Also, since the time span of our in vivo assay was relatively short, the effect of necroptosis to long-term tumor growth was not accessed. Therefore, future research needs to delve deeper into the mechanism by which necroptosis affects the growth of HNSCC.

ISG15 has been recognized by multiple previous studies as one of the highly upregulated genes and a potential biomarker in head and neck cancer [30–32], yet its involvement in HNSCC tumorigenesis is barely understood. Here, we demonstrated that ISG15 is programmatically released during necroptosis and further drives microenvironment reprogramming and lymphatic metastasis via RAGE. Since necroptosis is widespread in HNSCC [24], RAGE is widely distributed on human cells [86] and ISG15 is highly upregulated in HNSCC, the ISG15-RAGE axis may profoundly affect the tumorigenesis and progression of HNSCC. Our findings here shed light on mechanisms of the upregulation and functional involvement of ISG15 in HNSCC.

## Conclusion

In summary, our study revealed a previously unknown cGAS-ISG15-RAGE dependent reprogramming of the necroptotic microenvironment which converts the necroptotic stress into invasive force to facilitate HNSCC-cell dissemination. By demonstrating the programmatic production of ISG15 via necroptosis-cGAS orchestration and its downstream signaling through RAGE, we shed light on the unique role of ISG15 in HNSCC progression. Targeting these machineries may serve as potential therapeutic strategies to restore intratumoral survival stress and prevent lymphatic metastasis in HNSCC.

## Methods

### Immunohistochemistry

The immunohistochemical staining was conducted as previously described [24]. The scoring of IHC was performed following a previously described method [24]. Briefly, the extent was scored by the percentage of nuclear (for p-p65, p-STAT3 and Ki67) or cytoplasmic (for MLKL, cGAS and ISG15) or membrane (for RAGE, E-cad and N-cad) staining: 0 = less than 1%, 1 = 1–10%, 2 = 11–50%, 3 = 51–80%, and 4 = more than 80%. The intensity was scored as 1 = weak, 2 = moderate, and 3 = strong. Scoring was performed by two pathologists without prior knowledge of the sample information. The overall score is then calculated as (1 + intensity/3) * extent. For each Sect. 5 HPF (200x) was analyzed and the mean score was calculated as the final score.

The scoring of p-MLKL were conducted as we previously described [24]. In brief, the extent of necroptosis was scored as the percentage of p-MLKL positive cells: 0, no positive staining; 1, < 10% positive staining; 2, 10–30% positive staining; and 3, > 30% positive staining. The scoring was than binarized as Absent/Focal (score = 0–2) and Extensive (score = 3) for correlation and survival analysis.

### Cell culture and reagents

HNSCC cell lines SCC25, FaDu, colorectal adenocarcinoma cell line HT-29, pancreatic adenocarcinoma cell line BxPC-3 and AsPC-1, histiocytic lymphoma cell line U937, breast adenocarcinoma cell line MDA-MB-415 were purchased from American Type Culture Collection (ATCC, USA). SCC25 were cultured in DMEM/F12 1:1 (Gibco, USA) supplemented with 10% fetal bovine serum (FBS, Gibco, USA) and 400 ng/ml hydrocortisone (Solaria, Beijing, China). FaDu was cultured in MEM (Gibco, USA) supplemented with 10% FBS. BxPC-3, AsPC-1 and U937 were cultured in RPMI 1640 (Gibco, USA) supplemented with 10% FBS. HT-29 was cultured in McCoy’s 5a medium supplemented with 10% FBS. MDA-MB-415 was cultured in L-15 medium supplemented with 15% FBS, 10mcg/ml insulin and 10 mcg/ml glutathione.

Human recombinant TNF-α was purchased from PeproTech (USA). Smac mimetic AT-406 (SM-406), pan-caspase inhibitor zVAD-fmk, RIP1 inhibitor Necrostain-1 (Nec-1), RIP3 inhibitor GSK’872, and MLKL inhibitor Necrosulfonamide (NSA), cGAS inhibitor G150 and STING inhibitor C-170 were purchased from Selleck (Selleckchem, Houston, TX, USA). Human recombinant His6-ISG15 (Cat# UL-510), human recombinant GST-ISG15 (Cat# UL-600) and human recombinant ISG15 (Cat# UL-601) were purchased from R&D system (USA). Recombinant human HMGB1 (His Tag were purchased from Sino Biological (China). Small molecular inhibitor BAY 11-7082 (Cat# HY-13453), Stattic (Cat# HY-13818), FPS-ZM1 (Cat# HY-19370) were purchased from MCE (USA).

Antibodies against p65 (Cat# 8242), p-p65 (Cat# 3033), STAT3 (Cat# 9139), p-STAT3 (Cat# 9145), p-STAT5 (Cat# 931), MMP-9 ((Cat# 13667), E-cadherin (Cat# 3195), N-cadherin (Cat# 13116), Vimentin (Cat# 5741), ISG15 (Cat# 2758), IFIT1 (Cat# 1469), cGAS (Cat# 83623, #79978), STING (Cat# 13647), IRF3 (Cat# 11904), p-STING (Cat# 50,907), p-TBK1 (Cat# 5483), p-IRF3 (Cat# 37829), p-H2AX (Cat# 9718), Toll-like receptor 2 (Cat# 12276) were purchased from Cell Signaling Technology (USA). Antibodies against MLKL (Cat# ab184718), p-MLKL (Cat# ab187091), IL-6 (Cat# ab233706), ISG15 (Cat# ab285367), CD11a (Cat# ab52895), HMGB1 (Cat# ab79823) were purchased from Abcam (UK). Antibodies against IFIT2 (Cat# sc-390724), IFIT3 (Cat# sc-393512), RAGE (Cat# sc-365154) were purchased from Santa Cruz (USA). Antibodies against GST (Cat# 66001-2-Ig), His (Cat# 66005-1-Ig) were purchased from Proteintech (USA). Anti-GAPDH (CWBio Cat# CW0100M) antibody was purchased from CWBIO (China).

### Induction of cell death

The induction of apoptosis, necroptosis and passive necrosis were performed as previously described [24]. Briefly, combined treatment of TNF-α + Smac mimetic + zVAD-fmk (TSZ) was used to induce necroptosis, while TNF-α + Smac mimetic (TS) was used to induce apoptosis. Cells were pretreated with zVAD-fmk and Smac mimetic for 1 h. Then TNF-αwas added and treated cells for indicated time. DMSO was used as vehicle control. Classic freeze–thaw cycle (FT) was used to induce passive necrosis [7, 9, 64]. For inhibition of necroptosis, Necrostatin-1(Nec-1) were added together with S and Z before stimulation with TNF-α.

### Transwell migration and invasion assay

Transwell migration and invasion assay are performed as previously described [24, 83, 87]. Briefly, 6.5-mm-diameter polycarbonate filters (8-μm pore size) and 24-well plates were used. For invasion assay, the filter was pre-coated with 1:20 diluted Matrigel (BD biosciences) for 2 h. Cells received indicated treatments were resuspended in 200ul serum-free medium and seeded in the top chamber and the bottom chamber was filled with medium containing 10% FBS. Cells were allowed to migrate for 24–48 h. The cells were fixed with 4% paraformaldehyde and stained by crystal violet. Five images per chamber were taken using inverted microscope under 100 × magnification (Axio, ZEISS, Germany) and the migrated cells were counted using ImageJ software.

### PI staining

Propidium iodide (PI, BD Biosciences, USA) staining used for analysis of cell death rate as previously described [24]. Briefly, cells were harvested, washed twice with pre-cooled PBS and stained with PI solution for 5 min. The percentage of PI-positive cells was analyzed by CytoFlex (Beckman Coulter, USA).

### Western blotting

Western blotting was performed as we previously described [24]. Briefly, cells were lysed in RIPA buffer (Beyotime, China). For released proteins, cultural supernatants were concentrated using AmiconUltra (Millipore, USA) centrifugal filters. Protein samples were quantified by BCA protein assay kit (CWBIO, China). 20ug proteins (10ug for proteins from the supernatants) were loaded for SDS-PAGE gel electrophoresis and then transferred to polyvinylidene fluoride (PVDF) membranes (Millipore, USA). 2% skim milk or bovine serum albumin (BSA) were used for blocking unspecific bindings. The membranes were then incubated with primary antibodies at 4℃ overnight and then with secondary antibodies (EMAR, China) for 1 h at room temperature. Then the membranes were incubated in Immobilon ECL Ultra Western HRP Substrate (Millipore, USA) and the bands were detected using GeneGnome XRQ system (Syngene, USA).

### Dox-inducible necroptosis model

Tet-on lentivirus carrying full length human *MLKL* with phospho-mimetic mutation at T357E/S358D was generated by Hanbio Technology (Shanghai, China) using pHBLV-TetOn-SV40-Puro-TRE3GS-MCS lentiviral vector. SCC25 and FaDu cells were infected and screened for stably transfected cells (MLKL-25/MLKL-FD) as previously described [24]. Dox-induced MLKL expression was validated by western blotting and qRT-PCR. For knockdown of ISG15 in this dox-inducible necroptosis model, MLKL-FD and MLKL-25 cells were further infected with shISG15 lentivirus and screened for stably transfected cells (shISG15-MLKL-FD/25).

### Quantitative real-time PCR (qRT-PCR)

qRT-PCR was performed as previously described [24]. Briefly, total RNA was extracted using RNAzol (Molecular Research Center, Inc, USA) agent and quantified using NanoDrop2000. RNA reverse transcription process was performed using HiScript III RT SuperMix for qPCR kit (Vazyme, China) and qRT-PCR was performed using ChamQ SYBR qPCR Master Mix kit (Vazyme, China). Primer sequences are shown in Tables 2, 3.

**Table 2:** Primers for qRT-PCR

Gene name	Primer sequence
*CXCL1*	Forward: 5′-ATCATTGTGAAGGCAGGGGA-3′ Reverse: 3′-GCCCCTTTGTTCTAAGCCAGA-5′
*CXCL8*	Forward: 5′-GAGCCAGGAAGAAACCACCG-3′ Reverse: 3′-GGTCCACTCTCAATCACTCTCA-5′
*CXCL2*	Forward: 5′-ATCAATGTGACGGCAGGGAA-3′ Reverse: 3′-GCTCTAACACAGAGGGAAACAC-5′
*MMP9*	Forward: 5′-TGCGTCTTCCCCTTCACTTT-3′ Reverse: 3′-ACATAGGGTACATGAGCGCC-5′
*PLAU*	Forward: 5′-CCGCTTTCTTGCTGGTTGTC-3′ Reverse: 3′-CTATTGTCGTTCGCCCTGGT-5′
*BIRC3*	Forward: 5′-GGCTAGTCCCTTTTCTTCCCCATT-3′ Reverse: 3′-CAGCACGAGCAAGACTCCTTT-5′
*IL1A*	Forward: 5′-ACTGCCCAAGATGAAGACCAA-3′ Reverse: 3′-TTAGTGCCGTGAGTTTCCCA-5′
*IL6*	Forward: 5′-CCTCACCCTCCAACAAAGAT -3′ Reverse: 3′-GCCTCAGACATCTCCAGTCC -5′
*ICAM1*	Forward: 5′-TCAGAGATTACCCAGTGAGGC-3′ Reverse: 3′-CAGGACAAGAGGACAAGGCA-5′
*MMP1*	Forward: 5′-CTGAGGGTCAAGCAGACATCAT-3′ Reverse: 3′-AATGGGAGAGTCCAAGAGAATGG-5′
*CDKN1A*	Forward: 5′-GGACACACAAGAAGAAGGGCA-3′ Reverse: 3′-AAGGTCGCTGGACGATTTGA-5′
*ACTB*	Forward: 5′-TCAAGATCATTGCTCCTCCTGAG-3′ Reverse: 3′-ACATCTGCTGGAAGGTGGACA-5′
*MLKL*	Forward: 5’-CAACCTGAAGTAACAGCGAGA-3’
	Reverse: 3’-GGCTAATGGGGAGATAGAAAA-5’
*ISG15*	Forward: 5’-GCGCAGATCACCCAGAAGAT-3’
	Reverse: 3’-GTTCGTCGCATTTGTCCACC-5’
*IFIT1*	Forward: 5’-CAGCCTAGAGGGCAGAACAG-3’
	Reverse: 3’-TTGCCAGGTCTAGATGAGCC-5’
*IFIT3*	Forward: 5’-GCTGAGTCCTGATAACCAATACG-3’
	Reverse: 3’-GGCAAGGAGACTTTTCCAAGG-5’
*IFNB1*	Forward: 5’-AGCACTGGCTGGAATGAGAC-3’
	Reverse: 3’-TCATGAGTTTTCCCCTGGTG-5’
*GAPDH*	Forward: 5′-GAACGGGAAGCTCACTGG-3′ Reverse: 3′-GCCTGCTTCACCACCTTCT-5′

**Table 3:** Primers for genomic PCR

Gene name	Primer sequence
*ND2*	Forward: 5’-AAATAAAATGACAGTTTGAACATACAA-3’
	Reverse: 3’-TAAAAGGGGAGATAGGTAGGAGTAGCG-5’
*CYTB*	Forward: 5’-CAACCAGTAAGCTACCCTTTTACCATC-3’
	Reverse: 3’-GCCCATTTGAGTATTTTGTTTTCAATT-5’
*GAPDH*	Forward: 5’-CTGGCATTTGCTGAACGCAT-3’
	Reverse: 3’-AGTGCAGCCAGGTCTAATTGT-5’
*ACTIN*	Forward: 5’-TCAAGATCATTGCTCCTCCTGAG-3’
	Reverse: 3’-ACATCTGCTGGAAGGTGGACA-5’

### Immunofluorescence

Treated cells were fixed with 4% paraformaldehyde for 30 min followed by membrane permeabilization by 0.1%Triton for 15 min. Goat serum was used to block nonspecific bindings for 30 min. Cells were incubated with primary antibody overnight at 4℃ and then with fluorescent secondary antibody for 1 h at room temperature. At last, nuclei were stained by DAPI and Antifade Mounting Medium (Beyotime, China) were added to prevent fluorescence quenching. Stained cells were visualized under the LSM980 laser scanning confocal microscope (Axio, ZEISS, Germany). Quantification was performed by calculating the percentage of positive staining cells in each field. For each group, at least 4 randomly selected fields are used for quantification. The quantification of colocalization was performed by analyzing Pearson’s correlation using Image-Pro Plus 6.0 software.

### Collection of DAMPs

After inducing SCC25 and FaDu with different treatments for 12 and 24 h respectively, culture medium was renewed to eliminate the stimulation of drugs. Cells were cultured for additional 12 h and the culture medium were collected. The conditioned medium was centrifuged under 2000 × g for 10 min to eliminate cell debris before using for further experiments. For collection of the DAMPs from accidental necrosis, cells were cultured for the same time and went through three times frozen-thaw (F/T) cycles and centrifuge to eliminate cell debris. For DAMPs collection in Dox-inducible necroptosis model, MLKL-25 and MLKL-FaDu cells were induced with 1ug/ml Doxycycline for 12 h before supernatant were collected.

### In vivo assay

For Dox-induce intratumoral necroptosis model, luciferase-containing FaDu cells (Luc-FD) and dox-inducible necroptosis cells (MLKL-FD/shISG15-MLKL-FD) cells were mixed at a ratio of 3:1 and reach a final concentration of 6 × 10^6^ cells/ml. The mixing ratio of 3:1 was determined based on our pre-experiment to ensure an adequate amount of dox-induced necroptosis in the xenograft tumor which largely mimic the extensive necroptosis we previously observed in the clinical specimens [24]. Then 50ul of cells were orthotopically inoculated into the BALB/c nude mice. Five days after inoculation, 0.2 mg/ml Doxycycline was added to the drinking water to induce intratumoral necroptosis. For the inhibition of RAGE, 1 mg/kg FPS-ZM1 were intraperitoneally injected per day from Day 5. Mice in other groups were injected with same volume of saline. At day 14, in vivo imaging (IVIS system, PerkinElmer, USA) was used to observe cervical lymphatic metastasis before the mice were sacrificed, and then the primary tumors and cervical lymph nodes were collected for further analysis.

For intratumoral DAMPs injection model, Luc-FD cells were inoculated into the BALB/c nude mice. DAMPs were collected as above mentioned and concentrated by AmiconUltra (Millipore, USA) centrifugal filter. At day 1, 5 and 10, each mouse was intratumorally injected with 50ul of DAMPs. At day 15, cervical lymphatic metastasis was observed by live imaging (IVIS system, PerkinElmer, USA). Mice were then sacrificed, and the primary tumors and cervical lymph nodes were collected for further analysis.

The tumor volume was calculated as: volume = long diameter × short diameter^2 × 1/2.

The cervical lymphatic metastasis was analyzed by HE and pan-CK staining.

### RNA-seq

SCC25 cells were treated by apoptotic (TS), necrotic (FT) and necroptotic (TSZ) DAMPs for 24 h, and total RNA were extracted using Trizol (Invitrogen, USA). The RNA-seq was performed by BGI (Shenzhen, China). Differential expression analysis was performed using the DESeq2(v1.4.5) with Q ≤ 0.05. GO (http://www.geneontology.org/) and KEGG (https://www.kegg.jp/) enrichment analysis of annotated different expression gene was performed by Phyper (https://en.wikipedia.org/wiki/Hypergeometric_distribution) based on Hypergeometric test. The significant levels of terms and pathways were corrected by Q value with a rigorous threshold (Q ≤ 0.05) by Bonferroni. Functional alterations were further evaluated by gene-set enrichment analysis (GSEA).

### Label-free proteomic analysis

The apoptotic (TS), necrotic (FT) and necroptotic (TSZ) DAMPs from SCC25 cells were collected as aforementioned. The label-free proteomic analysis was conducted under the assistance of Applied Protein Technology (Shanghai, China). The experimental procedures were as follow:

#### Protein extraction and digestion

SDT (4%SDS, 100 mM Tris–HCl, 1 mM DTT, pH7.6) buffer was used for sample lysis and protein extraction. Proteins was quantified with the BCA Protein Assay Kit (Bio-Rad, USA) following by tryptic digestion according to the filter-aided sample Preparation (FASP) procedure.

200 μg of proteins were incorporated into 30 μl SDT buffer (4% SDS, 100 mM DTT, 150 mM Tris–HCl pH 8.0). The detergent, DTT and other low-molecular-weight components were removed using UA buffer (8 M Urea, 150 mM Tris–HCl pH 8.0) by repeated ultrafiltration (Microcon units, 10 kD). Then 100 μl iodoacetamide (100 mM IAA in UA buffer) was added to block reduced cysteine residues and the samples were incubated for 30 min in darkness. The filters were washed with 100 μl UA buffer three times and then 100 μl 25 mM NH4HCO3 buffer twice. Finally, the protein suspensions were digested with 4 μg trypsin (Promega) in 40 μl 25 mM NH4HCO3 buffer overnight at 37 °C, and the resulting peptides were collected as a filtrate. The peptides of each sample were desalted on C18 Cartridges (Empore^™^ SPE Cartridges C18 (standard density), bed I.D. 7 mm, volume 3 ml, Sigma), concentrated by vacuum centrifugation and reconstituted in 40 µl of 0.1% (v/v) formic acid. The peptide content was estimated by UV light spectral density at 280 nm using an extinctions coefficient of 1.1 of 0.1% (g/l) solution that was calculated on the basis of the frequency of tryptophan and tyrosine in vertebrate proteins.

#### SDS-PAGE

20 µg of protein for each sample were mixed with 5X loading buffer respectively and boiled for 5 min. The proteins were separated on 12.5% SDS-PAGE gel. Protein bands were visualized by Coomassie Blue R-250 staining.

#### LC–MS/MS analysis

LC–MS/MS analysis was performed on a Q Exactive mass spectrometer (Thermo Scientific) that was coupled to Easy nLC (Proxeon Biosystems, now Thermo Fisher Scientific) for 60/120/240 min. The peptides were loaded onto a reverse phase trap column (Thermo Scientific Acclaim PepMap100, 100 μm*2 cm, nanoViper C18) connected to the C18-reversed phase analytical column (Thermo Scientific Easy Column, 10 cm long, 75 μm inner diameter, 3 μm resin) in buffer A (0.1% Formic acid) and separated with a linear gradient of buffer B (84% acetonitrile and 0.1% Formic acid) at a flow rate of 300 nl/min controlled by IntelliFlow technology. The mass spectrometer was operated in positive ion mode. MS data was acquired using a data-dependent top10 method dynamically choosing the most abundant precursor ions from the survey scan (300–1800 m/z) for HCD fragmentation. Automatic gain control (AGC) target was set to 3e6, and maximum inject time to 10 ms. Dynamic exclusion duration was 40.0 s. Survey scans were acquired at a resolution of 70,000 at m/z 200 and resolution for HCD spectra was set to 17,500 at m/z 200, and isolation width was 2 m/z. Normalized collision energy was 30 eV and the underfill ratio, which specifies the minimum percentage of the target value likely to be reached at maximum fill time, was defined as 0.1%. The instrument was run with peptide recognition mode enabled.

#### Identification and quantitation of proteins

The MS raw data for each sample were combined and searched using the MaxQuant 1.5.3.17 software for identification and quantitation analysis. Related parameters and instructions are as follows:

**Table:** 

Item	Value
Enzyme	Trypsin
Max missed cleavages	2
Fixed modifications	Carbamidomethyl (C),
Variable modifications	Oxidation (M),
Main search	6 ppm
First search	20 ppm
MS/MS Tolerance	20 ppm
Database	uniprot_Homo_sapiens_188433_20200217.fasta
Database pattern	Reverse
Include contaminants	True
protein FDR	≤ 0.01
Peptide FDR	≤ 0.01
Peptides used for protein quantification	Use razor and unique peptides
Time window (match between runs)	2 min
protein quantification	LFQ
min. ratio count	1

#### Bioinformatic analysis

For Bioinformatic analyses, Cluster 3.0 (http://bonsai.hgc.jp/~mdehoon/software/cluster/soft-ware.htm) and Java Treeview software (http://jtreeview.sourceforge.net) were used to performing hierarchical clustering analysis. CELLO (http://cello.life.nctu.edu.tw/) which is a multi-class SVM classification system, was used to predict protein subcellular localization. Protein sequences are searched using the InterProScan software to identify protein domain signatures from the InterPro member database Pfam. The protein sequences of the selected differentially expressed proteins were locally searched using the NCBI BLAST + client software (ncbi-blast-2.2.28 + -win32.exe) and InterProScan to find homologue sequences, then gene ontology (GO) terms were mapped, and sequences were annotated using the software program Blast2GO. The studied proteins were blasted against the online Kyoto Encyclopedia of Genes and Genomes (KEGG) database (http://geneontology.org/) to retrieve their KEGG orthology identifications and were subsequently mapped to pathways in KEGG. Enrichment analyses were applied based on the Fisher’ exact test, considering the whole quantified proteins as background dataset. Benjamin-Hochberg correction for multiple testing was applied to adjust derived *p*-values, only functional categories and pathways with *p*-values under a threshold of 0.05 were considered as significant.

### Gene knockdown by siRNA/shRNA

Small interfering RNAs were designed and synthesized by RiboBio (Guangzhou, China). The siRNA sequences are shown in Table 4. Additionally, siRNA targeting RAGE (Cat# sc-36374) and corresponding negative control (Cat# sc-37007) were purchased from Santa Cruz (USA). For siRNA transfection, Lipofectamine^®^3000 was used as transfection agent according to the user protocol.

**Table 4:** Sequences for siRNAs used in this study

siRNAs	Target sequence (5’–3’)
si-IFIT1	GGAAGAACATGACAACCAA
si-IFIT3	GGATAATCACCCAGAGAAA
si-ISG15	TCCTGGTGAGGAATAACAA
si-cGAS	CTAGCAACTTAATTGACAA
si-STING	CTGGCATGGTCATATTACA
si-IRF3	GTGGACCTGCACATTTCCA

shISG15 lentivirus were generated by GeneCopoeia (USA) using psi-LVRU6H lentiviral vector based on the si-ISG15 sequence. Lentiviral transfection was conducted as previously described [24].

### Co-immunoprecipitation (Co-IP)

Protein A/G Magnetic Beads (MCE, USA) were pre-washed twice with 0.5% PBST and then incubated with primary antibodies on a rotator for 2 h at 4 ℃. The beads were then washed four times with 0.5% PBST and incubated with protein samples at 4 ℃ overnight. After washed four times with 0.5% PBST, the beads were resuspended in 1 × SDS-PAGE loading buffer (Cwbio, China) and incubated at 99 ℃ for 10 min to elute the immunoprecipitates for western blotting analysis.

For sequential IP of His-ISG15 and biotinylated surface protein, FaDu cells were treated with recombinant His-ISG15 for 2 h, then the surface protein were biotinylated using EZ-Link Sulfo-NHS-SS-Biotin (Thermo Fisher Scientific, USA) according to the manufacturer’s protocol, un-biotinylated FaDu cells were used as control. Cells were then lysed and centrifuged to remove the debris. The cell lysates were then incubated with anti-His Magnetic Beads (Cat#HY-K0209, MCE, USA) at 4℃ overnight. Captured proteins were eluted by Glycine (0,15M, pH = 2.5) following by incubating with Streptavidin Magnetic Beads (Cat#HY-K0208, MCE, USA) at 4 ℃ for 4 h. The beads were resuspended in 1 × SDS-PAGE loading buffer (Cwbio, China) and incubated at 99 ℃ for 10 min to elute the immunoprecipitates for western blotting analysis.

### Molecular docking

The ligand-receptor docking between ISG15 and TLR1/2/RAGE/CD11a were conducted using Rosetta2020 (Cambridge, USA) software under the assistance of Shenzhen Shuli Tech Co., Ltd. (Shenzhen, China). Briefly, the protein conformations were extracted from Protein Data Bank (PDB), the PDB codes are as follow: 1lfa (CD11a), 1z2m (ISG15), 2z7x (TLR1/2), 3o3u (RAGE). Pretreatment of protein crystal structures included removing water molecules, original ligands, and other irrelevant protein conformations. Then global docking was performed by Rosetta 2020 and the snapshots were rendered by Pymol. For each pair of proteins, 10,000 conformations were generated. All conformations were then assessed by the InterfaceAnalyzer module of Rosetta 2020 and the conformations with packstate ≥ 0.65 and dG_separated/dSASAx100 ≤ −1.5 were selected. Finally, the conformation with the highest total score was selected as the most possible binding mode and was further visualized by Pymol and Ligplot software.

### Analysis of the cytosolic release of mitochondrial DNA and its binding with cGAS

SCC25 cells were treated for indicated time, and the nuclear, mitochondrial, and cytosolic fractions were isolated using Mitochondria Isolation Kit for Cultured Cells (Thermo Fisher Scientific, USA). DNA were then extracted from mitochondrial, cytosolic, and nuclear fractions using Quick-gDNA MiniPrep Kit (Zymo Research, USA). Genomic PCR and DNA electrophoresis were performed to analyze the cytosolic release of mtDNA. The primers were listed in Table 2.

For the PicoGreen-MitoTracker double staining, the mitochondrial DNA were stained by PicoGreen (Lumiprobe, USA) for 1 h and the mitochondria were stained by Mitotracker red CMXRos (Thermo Fisher Scientific, USA) for 15 min. The nuclei were stained by Hoechst 33342 and then cells were treated by DMSO/TSZ/TSZ + Nec-1 for 8 h. Treated cells were visualized and captured using LSM980 laser scanning confocal microscope (Axio, ZEISS, Germany), and the colocalization was analyzed by Image Pro Plus 6.0 software. For continuous imaging, cells were visualized and captured under PE Operetta CLS (PerkinElmer, USA).

To detect the binding between cGAS and mtDNA, SCC25 cells were treated by DMSO/TSZ for 8 h. The cytosolic fractions were isolated as aforementioned. Cytosolic cGAS were then pulled down by immunoprecipitation using Protein A/G magnetic beads (MCE, USA) as aforementioned. DNA were then directly extracted from the beads using Quick-gDNA MiniPrep Kit (Zymo Research). Genomic PCR and DNA electrophoresis were performed to detect cGAS-bound mtDNA.

For ethidium bromide (EB)-mediated mtDNA depletion, SCC25 cells were cultured in the presence of 450 ng/ml or 600 ng/ml EB for 4 days. Cells were then seeded in 6 well plate and cultured without EB for 24 h to be ready for downstream experiments. The depletion of mtDNA was validated by genomic PCR.

### Patients’ cohort and tissue samples

A total of 108 patients that were diagnosed as head and neck squamous cell carcinoma (HNSCC) and received radical surgeries between 2015 and 2020 at the Department of Oral and Maxillofacial Surgery, Hospital of Stomatology, Sun Yat-sen University, were included in this study (Table 1). All patients had no history of pre-operative chemotherapy or radiotherapy. Paraffin-embedded primary tumor tissues were used for immunohistochemical and immunofluorescent staining. Clinicopathological parameters and follow-up data for all the study participants were collected. The starting point for patients’ survival was the date of surgery. The endpoint for OS, RFS and PFS was the date of patients’ death, locoregional recurrence, and tumor progression (locoregional recurrence or lymphatic/distant metastasis), respectively. Clinical staging and histological grading were based on the 8th edition of UICC/AJCC TNM classification. This study is approved by the Ethical Committee of the Stomatological Hospital of Sun Yat-sen University (No. KQEC-2024-78-01). Informed consents were obtained from all the participants.

### Statistics

SPSS 20.0 software (SPSS, USA) and GraphPad Prism 9 (GraphPad Software, USA) were used for statistical analysis. Kruskal–Wallis’s test and Dunn’s multiple comparison test were used for comparison of the IHC scores of p-p65 and p-STAT3. Fisher’s exact test was used for comparison of metastatic rates. Unpaired student’s t-test was used for the comparison of two groups of quantitative data (mRNA relative expression, cell counts, etc.). One-way ANOVA was used for comparing multiple groups of quantitative data, Turkey’s multiple comparisons test was used for pairwise comparison between each group. Simple linear regression model was employed to analyze the correlation between the expression of two proteins. *p* < 0.05 was considered statistically significant. Quantitative data are showed as mean ± SD unless stated otherwise. For each experiment, data are representative of at least two replications, with similar results obtained.

For bioinformatic analysis, aside from those mentioned above, STRING software (http://string-db.org/) was used for protein–protein interaction (PPI) analysis and MCL clustering. GEPIA (http://gepia.cancer-pku.cn/index.html) and cBioPortal (http://www.cbioportal.org/) were used for analyses of human HNSCC datasets derived from The Cancer Genome Atlas (TCGA: http://cancergenome.nih.gov/). The OmicShare online platform (https://www.omicshare.com) was used for hierarchical clustering, GSEA and visualization of results.

### Supplementary Information

Supplementary material 1. This file includes all the supplementary figures and legends that are mentioned in the main text.

Supplementary material 2. This video shows the programmatic production of ISG15 during necroptosis that mentioned in Figure. 6J.

Supplementary material 3. This video shows the programmatic production of ISG15 during necroptosis that mentioned in Figure. 6J.

## Data Availability

Further information and requests for resources and reagents should be directed to and will be fulfilled by the Lead Contact, Liang Yujie (liangyj35@mail.sysu.edu.cn). The raw data of RNA-seq have been deposited in the NCBI Sequence Read Archive (SRA), the accession code is PRJNA848011. The proteomic raw data have been deposited in iProX, the PXD code is PXD035379. All data supporting the findings of this study are available from the corresponding authors upon reasonable request.

## Abbreviations

HNSCC: Head and neck squamous cell carcinoma
MLKL: Mixed lineage kinase domain-like
RIP1: Receptor interacting protein kinase 1
RIP3: Receptor interacting protein kinase 3
DAMPs: Damage associated molecular patterns
cGAS: Cyclic GMP-AMP synthase
STING: Stimulator of interferon genes
NF-κB: Nuclear factor kappa-B
STAT3: Signal transducer and activator of transcription 3
IFIT1: Interferon induced protein with tetratricopeptide repeats 1
IFIT2: Interferon induced protein with tetratricopeptide repeats 2
IFIT3: Interferon induced protein with tetratricopeptide repeats 3
ISG15: ISG15 ubiquitin like modifier
TLR2: Toll-like receptor 2
RAGE: Receptor of advanced glycosylation end-product
HMGB1: High mobility group box 1 protein
