# Supplementary material for: cGAS-ISG15-RAGE axis reprogram necroptotic microenvironment and promote lymphatic metastasis in head and neck cancer
Source: Exp Hematol Oncol. 2024 Jun 26;13:63. doi: 10.1186/s40164-024-00531-5 (PMC11200990; doi:10.1186/s40164-024-00531-5)
Supplement: Supplementary file 1 — Supplementary material 1. This file includes all the supplementary figures and legends that are mentioned in the main text. [file 40164_2024_531_MOESM1_ESM.pdf]

Supplementary Figure & Legends

Supplementary Figure 1

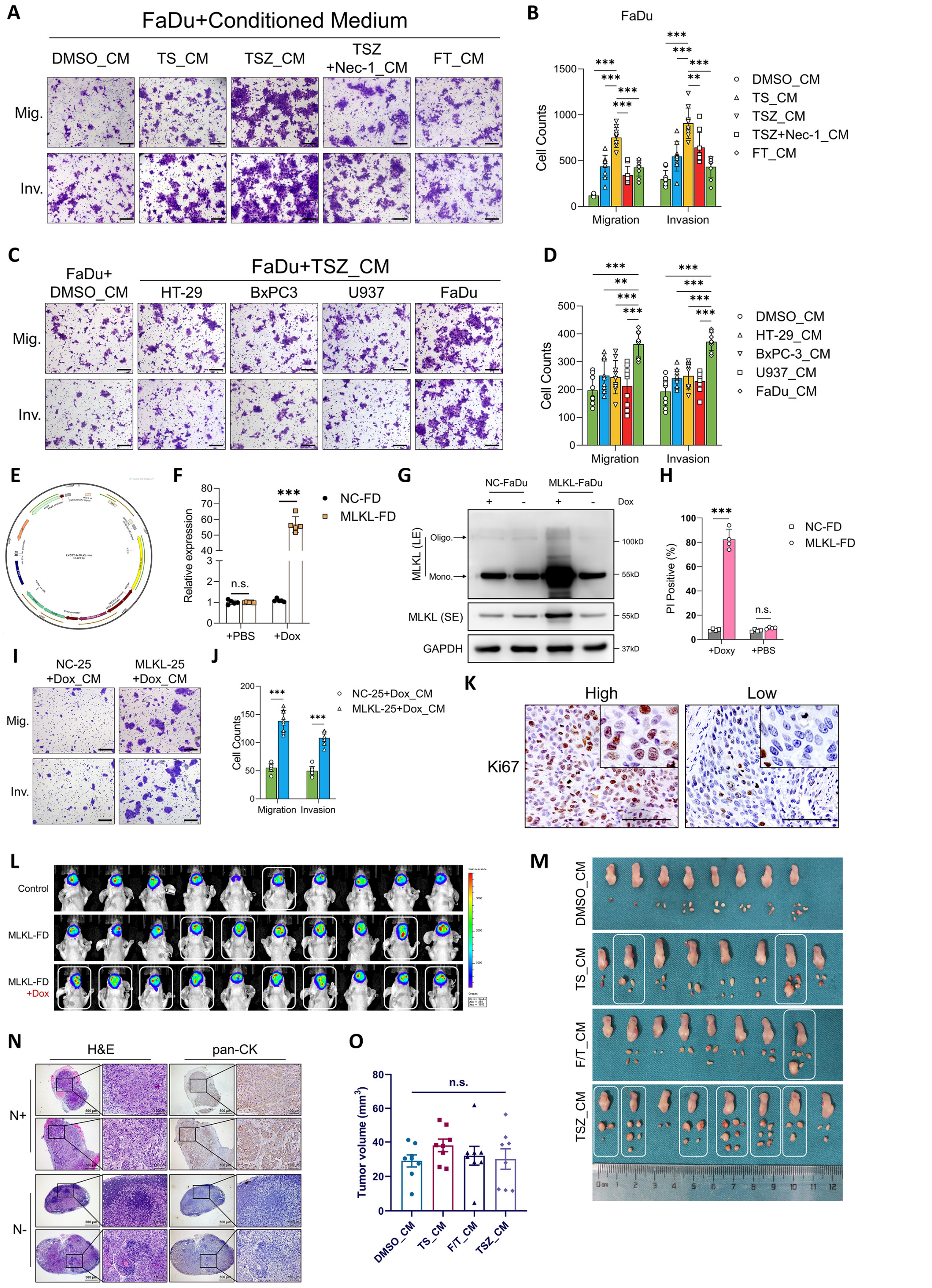

Supplementary Fig. 1 (A, B) Conditioned medium (CM) derived from TS (apoptosis), TSZ (necroptosis), FT (necrosis), TSZ+Nec-1 (inhibited necroptosis) and DMSO (vehicle control) treatments were collected and used to treat FaDu cells for 24 hours, and the migration and invasion

were analyzed by Transwell assays. Scale bar=200μm, n=9. (C, D) FaDu cells were treated by necroptotic DAMPs derived from HT-29, BxPC3, U937 and FaDu cells, or CM derived from DMSO treated cells. Tumor-cell migration and invasion were analyzed by Transwell assays (scale bar=200μm, n=9). (E) Schematic of tet-on lentiviral vector. SCC25 cells stably transfected with tet-on-MLKL(T357E/S358D) lentivirus (MLKL-25) and negative control virus (NC-25) were induced by 1ug/ml Doxycycline for 8 hours, the expression of MLKL were detected by qRT-PCR (F) and Western blotting (G). NC-25 and MLKL-25 were induced by Dox for 12 hours, PI staining was used to detect cell death rate (H). (I, J) Transwell migration and invasion assays of SCC25 cells treated by Dox-induced necroptotic DAMPs. Scale bar=200μm, n=9. (K) Representative of high- and low-Ki67 staining in xenograft tissues. Scale bar=100μm. (L) Live-imaging photos corresponding to the tissue samples presented in Fig. 2F. White frame indicated the mice with pathologically validated lymphatic metastasis. (M) Primary and cervical lymph nodes samples collected in the DAMPs injection assay. Samples are corresponding to the live-imaging photos presented in Fig. 2J. (N) Representative H&E and pan-CK stained images of metastatic and non-metastatic lymph nodes collected in the DAMPs injection assay. (O) Comparison of the volume of primary tumors from the DAMPs injection assay. Data are shown as mean ± SD unless stated otherwise. * *p-value*<0.05, ** *p-value*<0.01, *** *p-value*<0.001. Abbreviations: LE, long exposure; SE, short exposure.

Supplementary Figure 2

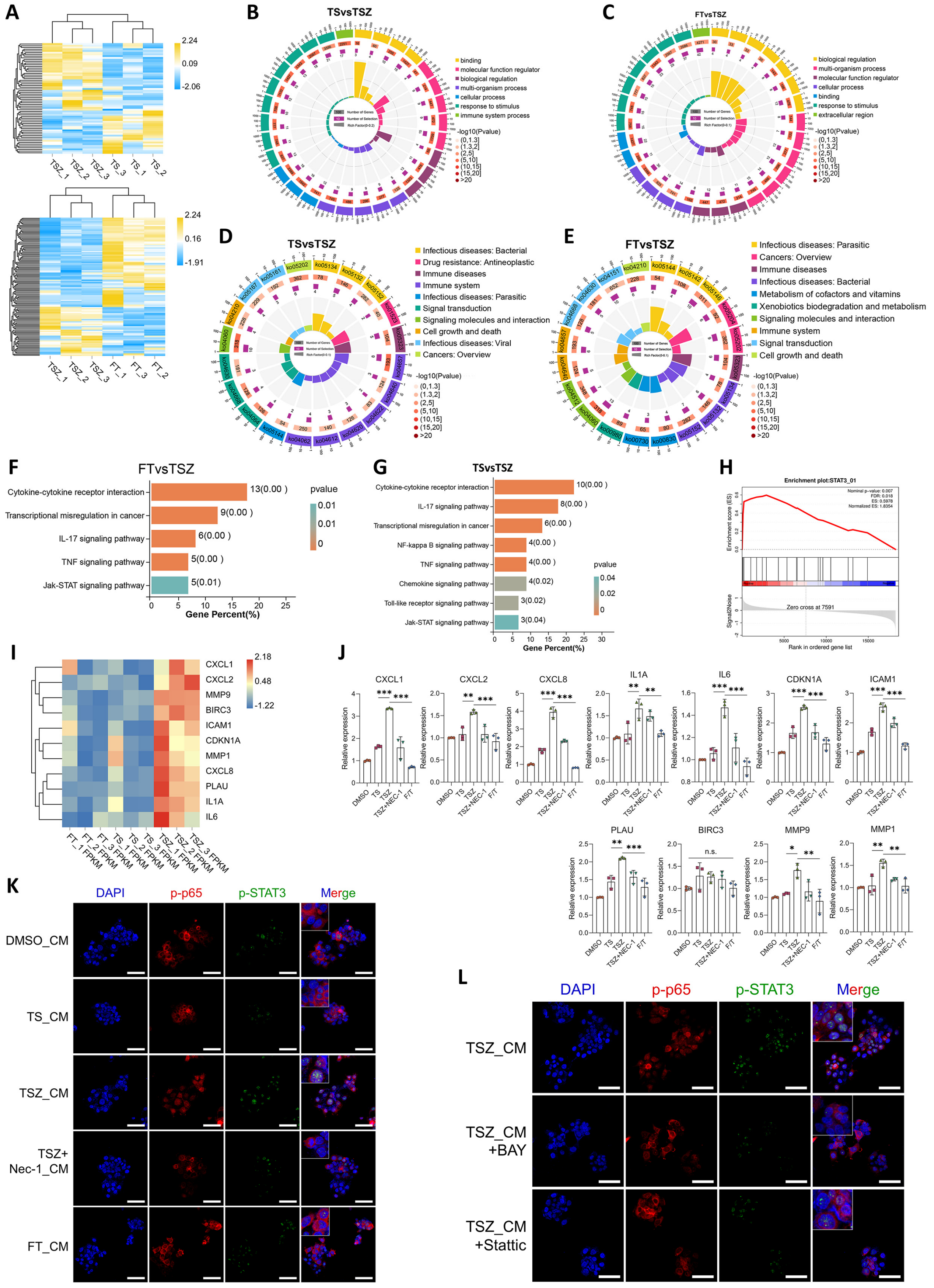

Supplementary Fig. 2 (A) Hierarchical clustering analysis showing differential expression patterns between TS and TSZ group (top panel) or FT and TSZ group (bottom panel). (B, C) Top 30 of enriched gene sets from GO enrichment analysis, the corresponding level 2 GO terms were shown on the right. (D, E) Top 20 of enriched gene sets and (F, G) the representative cytokine-related

signaling pathways from KEGG enrichment analysis. (H) GSEA results from c3_transcription factor targets databases. (I) Expression profile of selected cytokines/chemokines and proliferation-, migration- and invasion-related genes and (J) validations by qRT–PCR. FaDu cells were treated by (K) different CMs or (L) TSZ_CM±BAY 11-7082/Stattic for 24 hours. Immunofluorescence were performed to analyze the nuclear translocation of p-p65 and p-STAT3. Scale bar=100um. * *p-value*<0.05, ** *p-value*<0.01, *** *p-value*<0.001.

Supplementary Figure 3

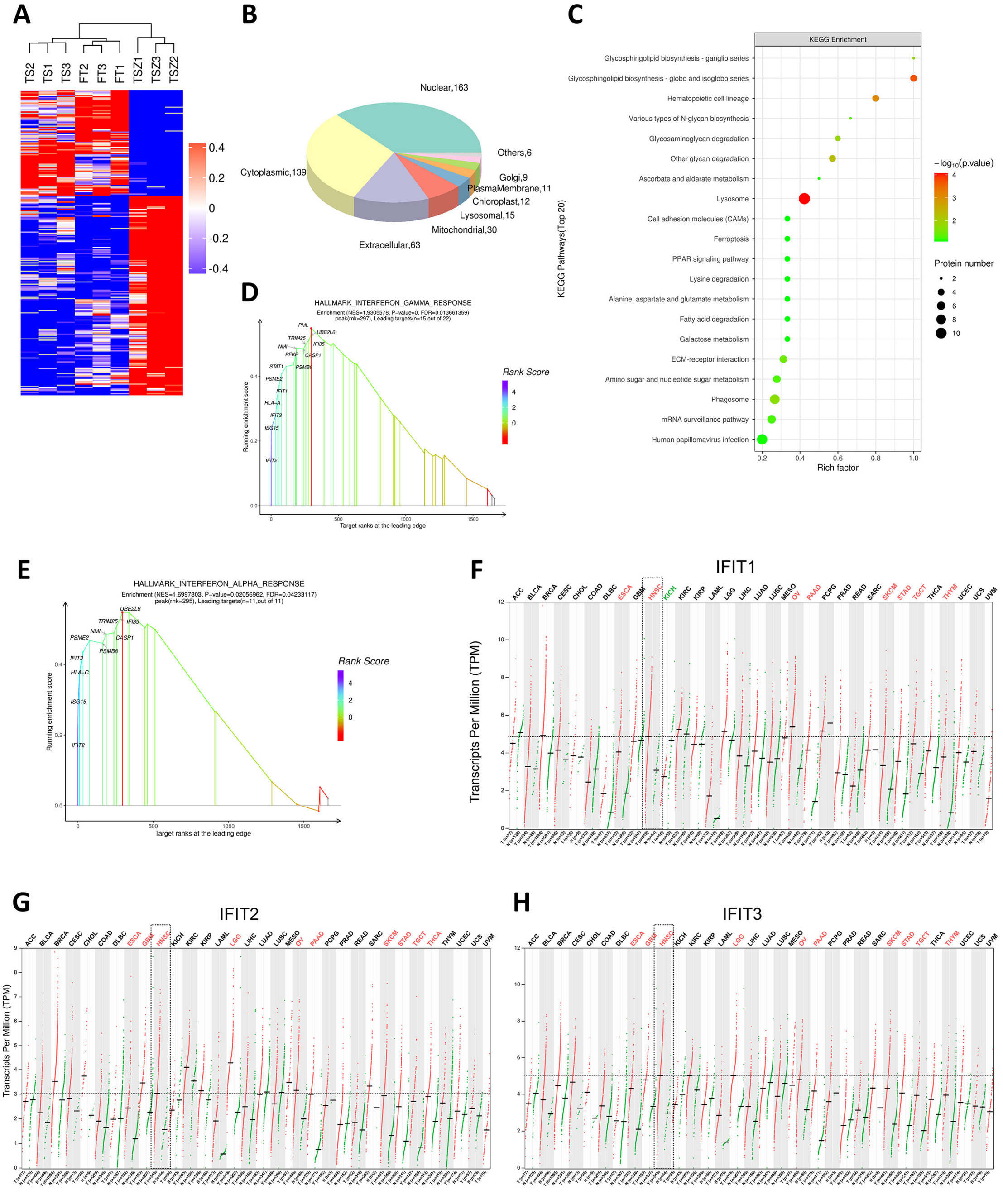

Supplementary Fig. 3 (A) Hierarchical clustering showing differential protein composition between TSZ and TS/FT group. (B) Subcellular locations annotation of differential proteins in TSZ group. (C) Top 20 enriched pathways from KEGG enrichment analysis of the differential proteins in necroptotic (TSZ) DAMPs. (D, E) Gene-set enrichment analysis (GSEA) showing the upregulation of interferon response in TSZ group. (G) Expression profile of IFIT1, IFIT2 and IFIT3 across various cancer types were plotted from TCGA database using GEPIA (http://gepia.cancer-pku.cn/index.html).

Supplementary Figure 4

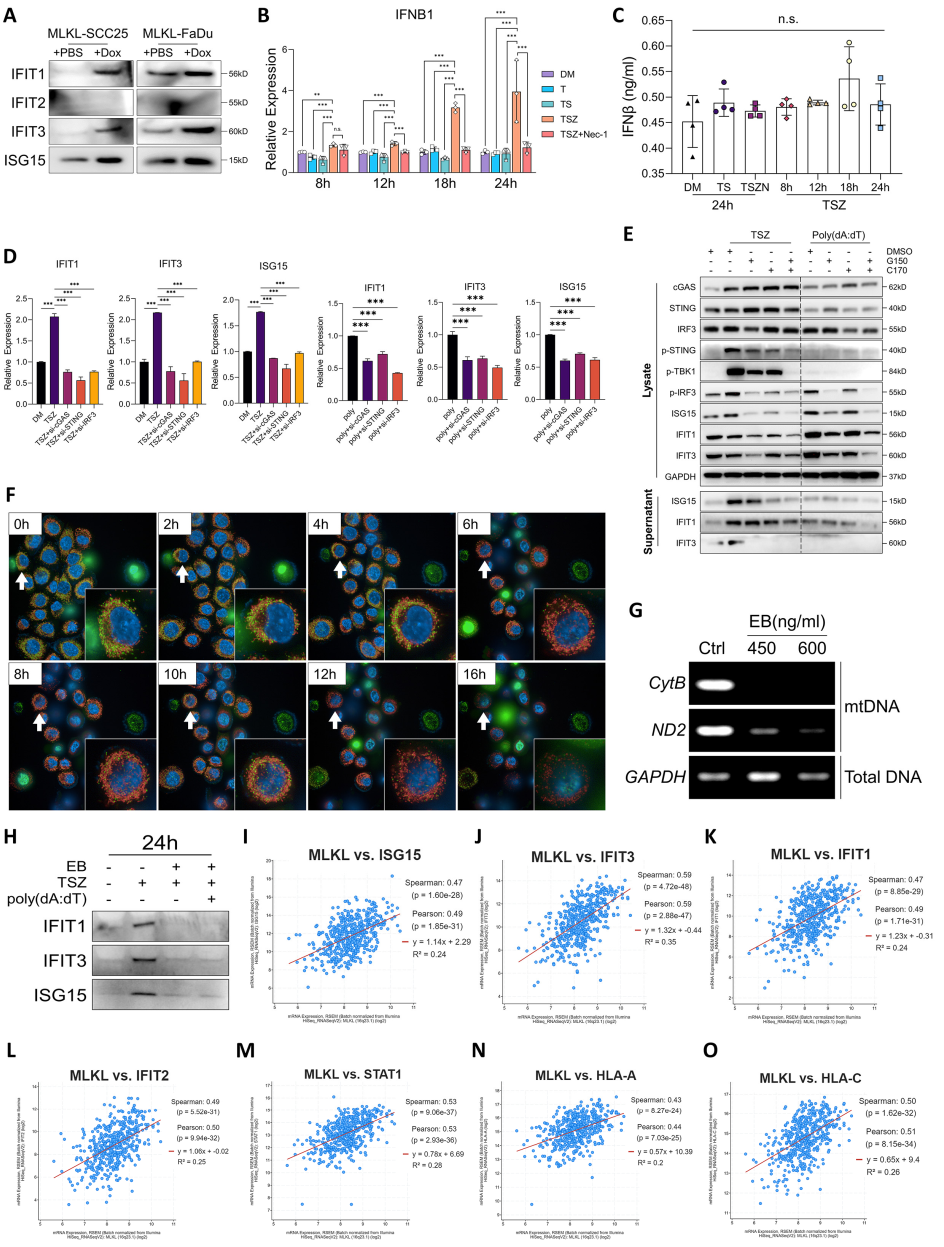

Supplementary Fig. 4 (A) Necroptosis was induced by Dox. The release of IFIT1, IFIT2, IFIT3 and ISG15 in the supernatants were detected by western blotting. SCC25 cells were treated for 8, 12, 18 and 24 hours, the transcription and secretion of IFNB1 were analyzed by (B) qRT-PCR and (C) ELISA. (D) SCC25 cells were pre-transfected with siRNA targeting cGAS, STING and IRF3 respectively, then cells were treated with TSZ or poly(dA:dT) for 12 hours. The mRNA level of IFIT1, IFIT3 and ISG15 were detected by qRT-PCR. (E) SCC25 cells were pre-treated with cGAS and/or STING inhibitor G150 and C170, followed by treatment of TSZ or Poly(dA:dT) for 12 hours. Protein-level alterations in cell lysate and supernatants were detected by western blotting. (F) SCC25 cells were pre-stained with Picogreen, MitoTracker and Hoechst 33342, followed by treatment with TSZ. Cells were continuously observed for mtDNA release using PE Operetta CLS (PerkinElmer, USA) imaging system. Images were captured every hour. (G) SCC25 cells were cultured in the presence of 450ng/ml or 600 ng/ml EB for 4 days. Genomic PCR was used to validate the depletion of mtDNA. (H) SCC25 and mtDNA-depleted SCC25 cells were treated by TSZ with or without poly(dA:dT) for 24 hours. The release of IFIT1, IFIT3 and ISG15 in the supernatant were detected by western blotting. (I-O) Co-expression of *MLKL* and *ISG15, IFIT1, IFIT2, IFIT3, STAT1, HLA-A, HLA-C* in the HNSCC dataset from TCGA database were analyzed using cBioPortal (https://www.cbioportal.org/).* *p-value*<0.05, ** *p-value*<0.01, *** *p-value*<0.001.

Supplementary Figure 5

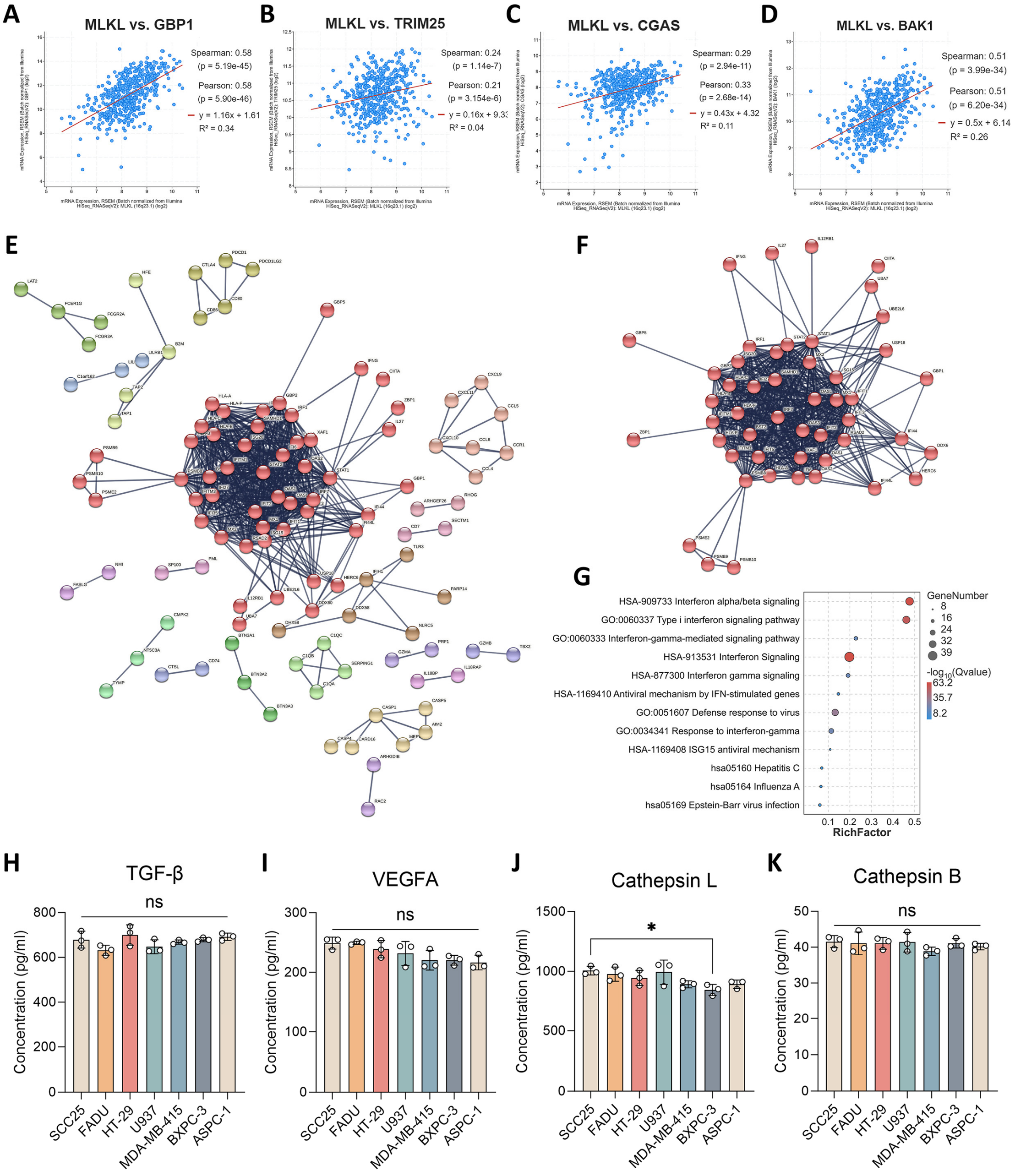

Supplementary Fig. 5 (A-D) Co-expression of *MLKL* and *GBP1, TRIM25, CGAS, BAK1* in the HNSCC dataset from TCGA database were analyzed using cBioPortal (https://www.cbioportal.org/). (E, F) The top 200 MLKL-co-expressed genes were analyzed by STRING for PPI and followed by MCL clustering. (G) Enrichment analyses were then performed to the biggest cluster (F). (H-K) Tumor cells with various histologic origins were treated by TSZ for 24 hours, the release of TGF-β, VEGFA, Cathepsin L and Cathepsin B in the supernatant were detected by ELISA. * *p-value*<0.05.

Supplementary Figure 6

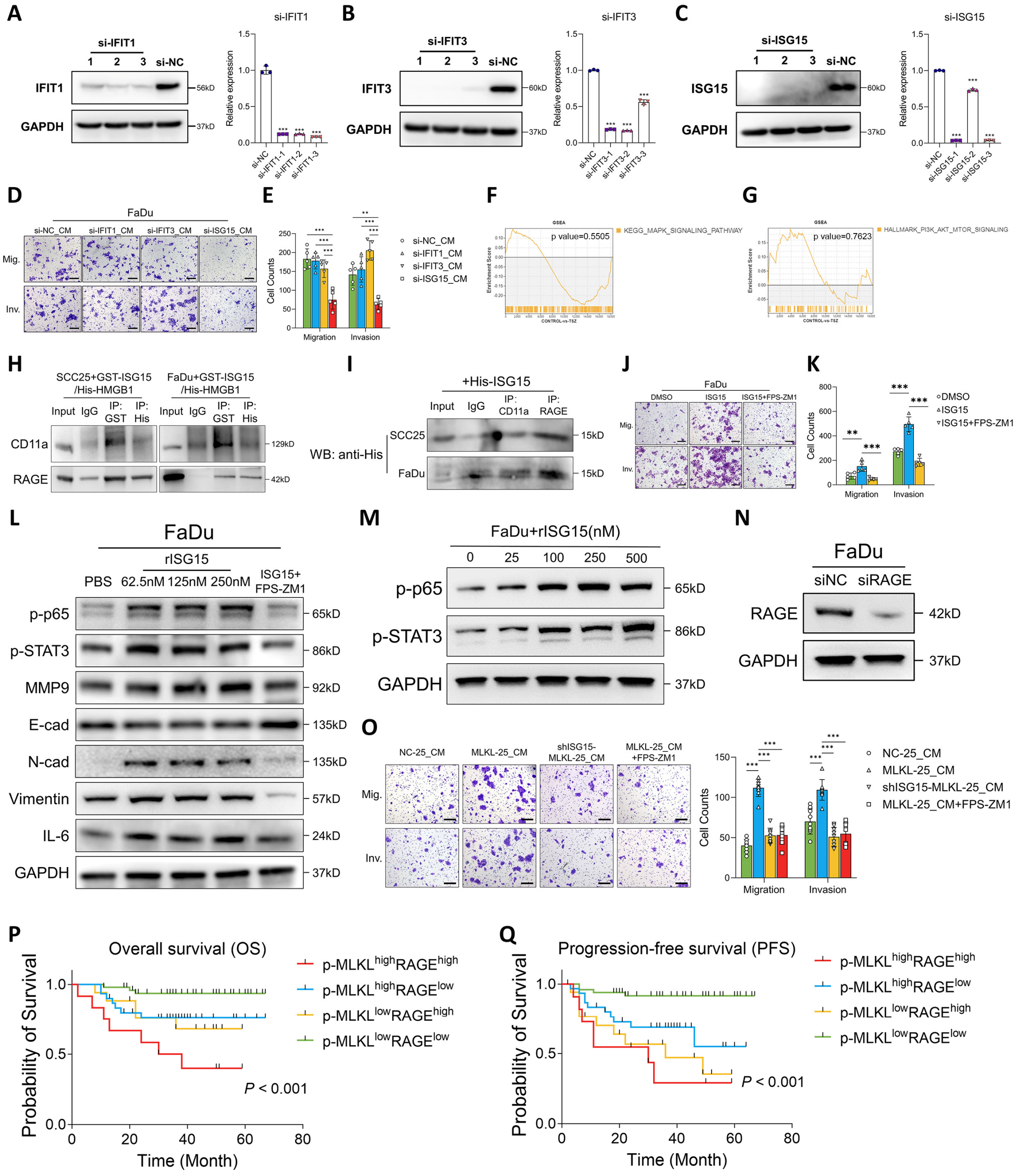

Supplementary Fig. 6 (A-C) Western blotting and qRT-PCR validating of the knockdown-efficiency of siRNAs targeting IFIT1, IFIT3 and ISG15. (D, E) FaDu cells were treated by IFIT1/IFIT3/ISG15-depleted necroptotic DAMPs for 24 hours. Transwell assays were used to analyze tumor-cell migration and invasion. Scale bar=200μm, n=5. (F, G) GSEA shows that MAPK and PI3K-Akt signaling were not enriched in necroptotic DAMPs (TSZ) treated cells. (H) SCC25 and FaDu cells were treated with either recombinant His-HMGB1 or GST-ISG15 for 2 hours. His-HMGB1 and GST-ISG15 were then pulled down by IP and their binding with RAGE and CD11a

were detected by western blotting. (I) SCC25 and FaDu cells were treated with recombinant His-ISG15 for 2 hours. CD11a and RAGE were then pulled down by IP and their binding with His-ISG15 were detected by western blotting. (J-L) SCC25 cells were treated with recombinant ISG15±FPS-ZM1 for 24 hours. Tumor-cell migration and invasion were analyzed by Transwell assays (J, K) and downstream protein alterations were analyzed by western blotting (L). Scale bar=200μm, n=5. (M) FaDu cells were treated by rISG15 in a gradient concentration (0-500nM) for 24hours, the activation of p65 and STAT3 were analyzed by western blotting. (N) FaDu were transfected with siRAGE or siNC for 48hours, the knockdown of RAGE was validated by western blotting. (O) Dox-induce necroptotic DAMPs were collected from MLKL-25 and shISG15-MLKL-25 cells. SCC25 cells were treated with NC-FD_CM, MLKL-FD_CM, shISG15-MLKL-FD_CM and MLKL-FD_CM+FPS-ZM1 for 24 hours. Tumor-cell migration and invasion were analyzed by Transwell assays. Scale bar=200μm, n=9. (P, Q) Kaplan–Meier survival analysis and log-rank test comparing the OS and PFS among p-MLKL^{high}RAGE^{high}, p-MLKL^{low}RAGE^{high}, p-MLKL^{high}RAGE^{low} and p-MLKL^{low}RAGE^{low} patients. * *p-value*<0.05, ** *p-value*<0.01, *** *p-value*<0.001.

Supplementary video 1&2. SCC25 transfected with promoter-ISG15-mCherry fused genes were treated with TSZ for 18 hours. The morphological changes and expression of ISG15-mCherry fusion proteins were continuously captured using PE Operetta CLS (PerkinElmer, USA). The videos were then exported in a frame rate of 20 fps.
